# Functional Expression of Recombinant *Candida auris* Proteins in *Saccharomyces cerevisiae* Enables Azole Susceptibility Evaluation and Drug Discovery

**DOI:** 10.3390/jof9020168

**Published:** 2023-01-27

**Authors:** Stephanie Toepfer, Michaela Lackner, Mikhail V. Keniya, Brian C. Monk

**Affiliations:** 1 Faculty of Dentistry, Sir John Walsh Research Institute, University of Otago, Dunedin 9016, New Zealand; 2 Institute of Hygiene and Medical Microbiology, Medical University of Innsbruck, Schöpfstrasse 41, 6020 Innsbruck, Austria; 3 Hackensack Meridian Health Center for Discovery and Innovation, Nutley, NJ 07110, USA

**Keywords:** *Candida auris*, yeast heterologous expression platform, azole resistance, multidrug resistance, emerging pathogens, amino acid substitutions, drug screening, human pathogenic fungi, candidiasis, candida infection

## Abstract

*Candida auris* infections are difficult to treat due to acquired drug resistance against one or multiple antifungal drug classes. The most prominent resistance mechanisms in *C. auris* are overexpression and point mutations in Erg11, and the overexpression of efflux pump genes *CDR1* and *MDR1*. We report the establishment of a novel platform for molecular analysis and drug screening based on acquired azole-resistance mechanisms found in *C. auris*. Constitutive functional overexpression of wild-type *C. auris* Erg11, Erg11 with amino acid substitutions Y132F or K143R and the recombinant efflux pumps Cdr1 and Mdr1 has been achieved in *Saccharomyces cerevisiae*. Phenotypes were evaluated for standard azoles and the tetrazole VT-1161. Overexpression of CauErg11 Y132F, CauErg11 K143R, and CauMdr1 conferred resistance exclusively to the short-tailed azoles Fluconazole and Voriconazole. Strains overexpressing the Cdr1 protein were pan-azole resistant. While CauErg11 Y132F increased VT-1161 resistance, K143R had no impact. Type II binding spectra showed tight azole binding to the affinity-purified recombinant CauErg11 protein. The Nile Red assay confirmed the efflux functions of CauMdr1 and CauCdr1, which were specifically inhibited by MCC1189 and Beauvericin, respectively. CauCdr1 exhibited ATPase activity that was inhibited by Oligomycin. The *S. cerevisiae* overexpression platform enables evaluation of the interaction of existing and novel azole drugs with their primary target CauErg11 and their susceptibility to drug efflux.

## 1. Introduction

The global incidence of invasive infections caused by non-albicans *Candida* species is an emerging problem. While nosocomial invasive candidiasis is most often caused by *Candida albicans,* an increased incidence of serious infections is due to non-*albicans* spp., such as *C. glabrata, C. tropicalis, C. parapsilosis,* and *C. auris* [1,2,3,4]. These pathogens, together with *Aspergillus fumigatus*, were recently highlighted by the World Health Organization as priorities for new research into diagnosis and treatment [5].

*C. auris* is a readily transmitted pathogen with a high mortality rate and resistance profiles that can compromise antifungal therapy and the operation of healthcare environments. First reported in 2009 as a clinical isolate from the external auditory canal of a Japanese patient [6], clinical isolates of *C. auris* were then obtained on three continents almost simultaneously, while a retrospective study identified a *C. auris* strain isolated in Korea in 1996 [7]. The first isolate was designated JCM 15448^T^ (alternative strain designations are CBS 10913^T^, DMS 21092^T^, and ATCC MYA-5001 [6,8]) and it is representative of clade II. Another clade II strain clonally related to JCM 15448^T^ is the haploid strain B11220 [9]. Both strains are susceptible to Fluconazole (FLC), Amphotericin B (AMB), and the echinocandins Anidulafungin and Caspofungin [6,7,10] but, compared to JCM 15448^T^, B11220 exhibits a 4-fold higher minimum inhibitory concentration (MIC) for FLC. This MIC difference may be due to a gene duplication encoding a putative phospholipid-translocating P-type ATPase and a putative cytochrome P450 [9].

Consistent with separate emergence inside continents, the current collection of *C. auris* strains shows greater genetic diversity between continents than within such geographic boundaries [7]. Genome sequencing and phenotypic analysis subsequently identified five major clades (Table 1), namely South Asian (I), East Asian (II), South African (III), South American (IV), and Iranian (V) [7,11,12]. A study in a neutropenic murine bloodstream infection model ranked the virulence of the *C. auris* clades as: clade IV > clade I > clade II = clade III [13]. In 2019, the US Centers for Disease Control and Prevention (CDC) announced *C. auris* as an urgent health threat due to: (1) misidentification and inappropriate patient management, (2) the number of outbreaks and their practical consequences in healthcare settings, and (3) multidrug-resistance phenotypes that make *C. auris* infections difficult to treat. The CDC suggested that novel antifungals and the use of combination therapy should be investigated for pan-resistant strains [14].

Clade-associated mutations conferring resistance to azole, echinocandin, and polyene antifungals have been found in *C. auris* (Table 1). Y132F is the most common substitution in the azole target *C. auris* Erg11 (CauErg11) and it has been found in clades I, IV, and V. While K143R is found in clades I and IV only, most of these mutations belong to clade I [7,12,15,16,17]. The Erg11 F126L point mutation has been reported exclusively in clade III [7,18]. Among triazole-resistant *C. auris* clinical isolates, *CDR1* and *MDR1* are highly expressed, with *CDR1* the main contributor to triazole resistance [20]. Gain-of-function mutations in *TACK1b*, the gene encoding the transcription factor driving Cau*CDR1* expression, were shown to be a principal cause of FLC resistance, with A640V the most prominent Tac1b substitution [22,23]. The CauCdr1 point mutation V704L has recently been found in azole-resistant *C. auris* isolates [31]. The N647T amino acid substitution in the CauMrr1 transcription factor involved in CauMdr1 expression has been identified in most clade III FLC-resistant isolates [24]. 

Up to 50% of systemic infections caused by *C. auris* are lethal [32,33,34]. Despite the development of novel triazoles, such as Isavuconazole (ISA) and the tetrazole VT-1161 (Oteseconazole), there is an urgent need to identify therapies for *C. auris* that selectively inhibit *Erg11* or efflux pumps without being compromised by mutations that either directly or indirectly affect the azole target enzyme or drug efflux.

This laboratory has implemented tools for biochemical and phenotypic analysis and structure-directed screens by expressing functional, recombinant full-length Erg11 from *Saccharomyces cerevisiae*, *C. albicans, C. glabrata* and *C. parapsilosis, Cryptococcus neoformans,* as well as the *S. cerevisiae* Pdr5 and *C. albicans* Mdr1 and Cdr1 drug efflux pumps, all expressed constitutively from the *PDR5* locus of a hypersensitive *S. cerevisiae* strain with seven drug efflux pumps removed [35,36,37,38,39]. This heterologous expression platform has the advantage that single protein targets of interest can be overexpressed in a hypersensitive background. This not only enables phenotypic and biochemical analysis of wild-type and mutant enzymes but also facilitates antifungal drug screens without complications, such as the background of multiple drug efflux pumps found in clinical isolates.

This report describes the overexpression and characterization of a functional hexahistidine-tagged wild-type CauErg11 enzyme, the CauErg11 Y132F and K143R mutants, as well as the drug efflux pumps CauMdr1 and CauCdr1, in the *S. cerevisiae* host strain ADΔΔ (Table S1). This platform enables whole-cell and biochemical measurement of drug susceptibility and sets the stage for the target-based screening of novel antifungals.

## 2. Materials and Methods

### 2.1. Strains and Cell Culture Reagents

All reagents used in culture media were purchased from Formedium^TM^ (Swaffham, Norfolk, UK) unless otherwise stated. *S. cerevisiae* was grown and maintained in Yeast Extract Peptone Dextrose medium (YPD, 1% yeast extract, 2% peptone, and 2% glucose). Synthetic defined (SD) histidine dropout medium (SD-His, 2% glucose, 0.77 g/L histidine dropout, 0.67% yeast nitrogen base without amino acids and solidified with 1.8% agar) was used to select transformants. Drug susceptibility testing assays used SD medium complemented with 0.79 g/L complete supplement mixture (CSM) and containing 10 mM MES (Roth, Karlsruhe, Germany) and 20 mM HEPES (Sigma-Aldrich, St. Louis, MO, USA), buffered with UltraPure^TM^ Tris (Invitrogen, Thermo Fisher Scientific, Waltham, MA, USA) to pH 6.8.

### 2.2. Chemicals and Drugs

Nile Red, 2-deoxy-D-glucose, Oligomycin (OM), and the antifungals FLC, Voriconazole (VRC), Posaconazole (POS), Itraconazole (ITC), and AMB were purchased from Sigma-Aldrich. ISA was purchased from BOC Sciences (Shirley, NY, USA), and micafungin (MFG) was a gift from Astellas Pharma Inc. (Osaka, Japan). The novel tetrazole antifungal VT-1161 and the Mdr1 inhibitor MCC1189 were synthesized by MicroCombiChem (Wiesbaden, Germany). Beauvericin was purchased from Cayman Chemicals (Ann Arbor, MI, USA). Desalted oligonucleotides were purchased from Integrated DNA Technologies (Coralville, IA, USA). Phusion Green Hot Start II High-Fidelity PCR master mix (2x) (Thermo Fisher Scientific, Waltham, MA, USA) was used for all PCR amplifications, except the use of TaKaRa Ex Taq DNA polymerase (TaKaRa Bio. Inc., Shiga, Japan) for colony PCR. NucleoSpin Gel and PCR clean-up kits (Macherey-Nagel, Düren, Germany) were used for DNA gel extraction and PCR purification. Genomic DNA was isolated using the Yeast DNA Extraction Kit (Thermo Fisher Scientific). Transformation of *S. cerevisiae* strains was performed using the Alkali-Cation^TM^ Yeast Transformation Kit (MP Biomedicals, LLC., Irvine, CA, USA). DNA sequence analysis of transformation cassettes and genes of interest in genomic DNA were performed at the Genetic Analysis Services facility (University of Otago, Dunedin, New Zealand). The presence of recombinant enzyme in Coomassie blue R250-stained SDS-PAGE separated protein bands was verified by mass spectrometry of tryptic fragments using an LTQ-Orbitrap XL mass spectrometer (Thermo Fisher Scientific) at the Centre for Protein Research (University of Otago, Dunedin, New Zealand).

### 2.3. Construction of Recombinant Strains

Recombinant strains were constructed to overexpress *C. auris ERG11*, *MDR1,* or *CDR1* from the *S. cerevisiae PDR5* locus. The host strain ADΔΔ (Y1857) has the *PDR3* transcriptional regulator gene and seven ABC transporters removed while the mutant gain-of-function transcriptional regulator *pdr1-3* gene enables constitutive expression from the *PDR5* locus [37]. Strains used or generated in this study are listed in supplementary Table S1.

The open reading frames of Cau*ERG11,* Cau*MDR1,* and Cau*CDR1* were amplified from genomic DNA of the clade II wild-type clinical isolate B11220 using the primers listed in Table S2. The transformation cassettes contained *CauERG11-6×His* (strain Y2767), *CauMDR1-6×His* (Y2765), or *CauCDR1-6×His* (Y2766), with each ORF containing a C-terminal GGR linker and hexahistine tag sequence, followed by the PGK transcription terminator and a downstream *HIS1* marker flanked by two LoxP sites. Each transformation cassette was bordered by *PDR5-*specific arms to enable homologous recombination at the *PDR5* locus of the host strain *S. cerevisiae* Y1857. The endogenous *ERG11* gene was also deleted in strain Y2767 using a disruption cassette with the recyclable selection marker *URA3* flanked by modified LoxP sites [40]. The *ERG11* mutants Y2768 (*CauERG11 Y132F-6×His*) and Y2769 (*CauERG11 K143R-6×His*) were constructed by incorporating the mutations Y132F and K143R, respectively, into *CauERG11-6×His* using the primers listed in Table S2**.** For this, the *HIS1* marker of *CauERG11-6×His* was deleted from the *PDR5* locus using the Cre-expressing plasmid pSH69 (Euroscarf, SRD, Germany). The galactose-inducible Cre recombinase mediated recombination between the two LoxP sites, allowing *HIS1* to be removed [41,42]. A mutant transformation cassette containing an *HIS1* marker was incorporated into the *PDR5* locus of the *CauERG11-6×His-*expressing strain via homologous recombination. The nucleotide sequences in the *PDR5* locus of transformants (Table S3) and in the endogenous *ERG11* locus of strain Y2767 were confirmed by DNA sequence analysis of genomic DNA.

### 2.4. Crude Membrane Preparation and Western Blot Analysis

Strains of interest were cultured in YPD at 30 °C with shaking at 200 rpm and harvested at OD_600nm_ = 6–8. Cell breakage was performed in a BeadBeater using 0.5 mm Zirconia/Silica beads (BioSpec, Bartlesville, OK, USA) and crude membranes isolated by differential centrifugation, as described previously [35]. The pellet of crude membranes was resuspended in 20% glycerol, 10 mM Tris, 0.5 mM EDTA, 0.5 mM PMSF, and pH 7.5 for storage. Protein content was estimated using the Lowry assay [43] with bovine serum albumin as standard. Crude membrane samples (20 µg protein) were separated by SDS-PAGE [44] on 8% Tris-Glycine polyacrylamide gels and stained with Coomassie blue R250 or used for Western blotting. The hexahistidine-tagged ScErg11, CauErg11, CauErg11 Y132F, CauErg11 K143R, CauMdr1, and CauCdr1 proteins, electro-transferred to Amersham^TM^ Hybond^®^ polyvinylidene difluoride (PVDF) membrane (Cytiva, MA, USA), were decorated with a mouse monoclonal anti-6×His tag antibody conjugated with horse radish peroxidase (Sigma-Aldrich), as previously described [38], and visualized using enhanced chemiluminescence (Clarity^TM^ Western ECL, Bio-Rad Laboratories Inc., Hercules, CA, USA). Gel imaging was performed using a ChemiDoc MP gel imager (Bio-Rad) and the relative expression of the recombinant His-tagged proteins on the Western blots was evaluated using Image Lab Software Version 6.1 (Bio-Rad).

### 2.5. Partial Purification of Hexahistidine-Tagged Proteins from Recombinant Strains

Cells were harvested from 2 L YPD cultures, crude membranes prepared, and CauErg11-6×His, CauErg11 Y132F-6×His, CauErg11 K143R-6×His, or CauMdr1-6×His partially purified by nickel–nitrilotriacetic acid (Ni-NTA) affinity chromatography, essentially as described by Monk et al. [35]. The crude membranes were extracted in pH 7.5 solubilization buffer (10% (*w*/*v*) glycerol, 250 mM NaCl, 20 mM Tris, 0.5 mM PMSF, 1 tablet cOmplete^TM^ Mini, EDTA-free Protease Inhibitor cocktail (Roche, Basel, Switzerland) per 50 mL plus 16 mM n-decyl-β-D-maltopyranoside (DM Anagrade, Anatrace Products, LLC., Maumee, OH, USA). The detergent-solubilized protein affinity purified by binding to 2 mL of Ni-NTA Agarose matrix (Qiagen, Hilden, Germany) per g of protein in a disposable Econo-Pac^®^ Chromatography column (Bio-Rad) was washed with 2 mM L-histidine and eluted with 100 mM L-histidine. The eluted protein was concentrated to 500 µL using Amicon^®^ Ultra–4 Centrifugal Filter Units with a 50 kDa cutoff (Merck Millipore Ltd., Tullagreen, Ireland). Samples (1 µL) were separated by SDS-PAGE to obtain Coomassie-blue-stained protein bands for mass spectrometry of tryptic fragments. Tryptic fragments from the SDS-PAGE separated CauCdr1-6×His-containing band in crude membranes were used for mass spectrometry. For protein identification, the obtained fragments were searched against the Swiss-Prot database, the sequence of interest, and *S. cerevisiae* database to exclude possible background contamination.

### 2.6. Antifungal Susceptibility Testing

Antifungal susceptibility profiles of recombinant strains were determined according to the European Committee on Antimicrobial Susceptibility Testing (EUCAST) of Yeasts version 7.3.2 with modifications. SD medium buffered to pH 6.8 was used instead of RPMI-1640 to provide the inoculum and growth conditions optimized for *S. cerevisiae*. The assays were carried out in flat-bottom 96-well plates (Greiner Bio-One Cellstar, Kremsmünster, Austria) containing 100 µL of antifungal in serial dilution in SD medium or 100 µL of SD medium with 0.5% DMSO for the no-drug control. The plates were seeded with 100 µL of SD pH 6.8 grown cells at an OD_600nm_ = 0.02 and incubated with shaking at 100 rpm under 75% humidity (Infors HT Minitron, Infors AG, Bottmingen, Switzerland) for 48 h at 30 °C. The OD_600nm_ was read using a Synergy 2 plate reader (BioTek, Winooski, VT, USA). For all antifungals, the MIC was read as ≥80% growth inhibition (MIC_80_) of the non-drug control after subtraction of the medium background. Three separate clones of each construct were tested in at least three biological replicates.

### 2.7. Nile Red Efflux Assay

Nile Red efflux was measured as previously described [45,46]. Cells were grown overnight at 30 °C in five mL of SD medium pH 6.8 with shaking at 200 rpm (Multitron, Infors AG, Bottmingen, Switzerland). A secondary culture in 40 mL of SD medium pH 6.8 was adjusted to OD_600nm_ = 0.5 and incubated for ~7 h until an OD_600nm_ ~2 was achieved. The pellet was washed 3 times with 10 mL of ice-cold phosphate-buffered saline (PBS) via centrifugation at 3900× *g* for 5 min and incubated on ice overnight. Cells were warmed to room temperature for 30 min then resuspended in 10 mL 50 mM HEPES buffered with 0.1M NaOH to pH 7.0. The cells were starved by incubation with 5 mM 2-deoxy-D-glucose (2-DOG) for 30 min at 30 °C with gentle agitation (50 rpm). Nile Red (7.5 µM final concentration) was added, and the cells were incubated for 30 min at 30 °C. The cells were washed twice with ice-cold HEPES buffer, adjusted in the HEPES buffer to OD_600nm_ = 10, and kept on ice for 2 h. Flat-bottom 96-well plates were prepared containing 50 µL of the HEPES buffer. A 100 µL sample of Nile-Red-loaded fungal cells was added to each well. Efflux pump activity was initiated by adding 50 µL 80 mM glucose while the addition of 50 µL of HEPES buffer served as a control. Fluorescence intensity was measured using a Synergy 2 plate reader at excitation wavelength 485/20 nm and emission wavelength 528/20 nm at 35 s intervals for 10 min, with 5 s shaking between readings. Nile Red fluorescence intensity was expressed as a percentage relative to the intensity at time T_0_.

### 2.8. ATPase Activity of the Recombinant Strain Overexpressing CauCDR1-6×His

The ATPase activity of recombinant strains overexpressing Cdr1 was measured in the presence and absence of OM, using crude membranes isolated from 40 mL YPD cultures, as previously described [47,48,49,50]. Crude membranes (0.1–0.2 µg of protein per well) were incubated with assay cocktail (50 mM Tris-HCl pH 7.5, 50 mM potassium nitrate, 0.2 mM ammonium molybdate, 5 mM sodium azide) and 6mM Mg-ATP in a total volume of 120 µL. The OM-sensitive component of the ATPase activity was determined by adding OM (0.027–20 µg/mL) to the assay cocktail. After incubation for 1 h at 30 °C, the reaction was stopped by adding 130 µL of development reagent (1% SDS, 1.6% sodium L-ascorbate, 1.2% ammonium molybdate in 6 M H_2_SO_4_). The amount of free phosphate was measured using a Synergy 2 plate reader at 750 nm with KH_2_PO_4_ as standard. The ATPase activity of crude membranes from the negative control strain Y2411, which lacks Cdr1, was subtracted from the activity of Cdr1-overexpressing strains.

### 2.9. Type II Azole Binding

The in vitro function of recombinant Erg11 in strains Y2767, Y2768, and Y2769 was analyzed in type II drug-binding studies. The concentration of functional wild-type enzyme was determined using a carbon monoxide (CO) binding assay [51]. Proteins of interest, partially purified by Ni-NTA affinity chromatography, were saturated by bubbling with CO before adding 1 mg sodium dithionite (Sigma-Aldrich). The reference contained the same amount of protein and dithionite but was not treated with CO. Difference spectra were determined in a Cary 1 Bio UV-visible spectrophotometer (Agilent Technologies, Santa Clara, CA, USA) using 10 mm path UV-transparent plastic cuvettes (GE Healthcare Life Sciences, Chicago, IL, USA). The concentration of cytochrome P450 was estimated using difference spectra obtained between 380 nm and 450 nm by applying an extinction coefficient of 91 mM^−1^cm^−1^ [52].

Type II binding of azoles was measured using difference spectra between 350 and 500 nm, with 1 µM enzyme titrated with POS, VRC, or ISA. The antifungals were dissolved in DMSO and added to the sample cuvette. The same amounts of DMSO were added to the reference cuvette, with the total amount of DMSO in the cuvette not exceeding 2%. The trough–peak difference in the spectra was used to plot the binding curves. The dissociation constant K_d_ was calculated using GraphPad Prism 9 software by applying a non-linear regression analysis (Levenberg-Marquardt method) and obtaining the best fit of the data for the Hill and Michaelis–Menten binding equations.

### 2.10. Statistical Analysis

Statistical analyses were performed with GraphPad Prism 9 (Graph Pad Software, LLC., San Diego, CA, USA).

## 3. Results

### 3.1. Biochemical Characterization of Recombinant Strains

**Figure 1:**
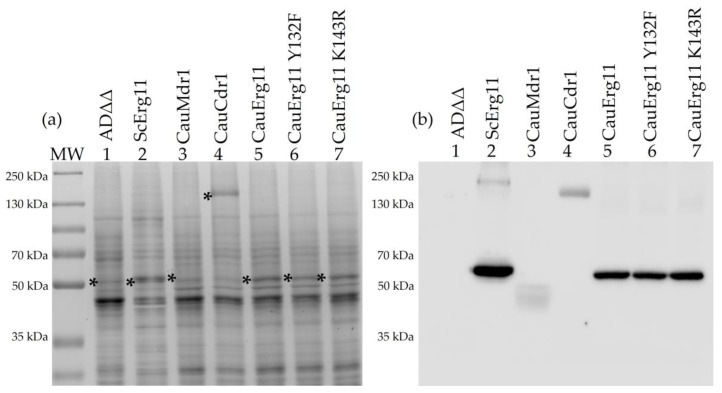
SDS-PAGE and corresponding Western blot of crude membrane preparations from recombinant strains. (**a**) Coomassie-Blue-stained SDS-PAGE of 20 µg crude membranes expressing recombinant *C. auris* Erg11, Mdr1, and Cdr1 proteins. Bands of interest are marked with an asterisk. (**b**) Western blots of 20 µg protein/lane decorated with mouse anti-6×His antibody and visualized using ECL. Lanes: (**a**,**b**) 1. ADΔΔ (Y1857), 2. ScErg11-6×His (Y941), 3. CauMdr1-6×His (Y2765), 4. CauCdr1-6×His (Y2766) 5. CauErg11-6×His (Y2767), 6. CauErg11 Y132F-6×His (Y2768), 7. CauErg11 K143R-6×His (Y2769). MW: 5 µL protein molecular-weight markers (PageRuler Plus Prestained Protein Ladder, Thermo Fisher Scientific, Waltham, MA, USA).

The *S. cerevisiae* strains generated in this study are listed in Table S1. The strains were constructed to constitutively overexpress recombinant hexahistidine-tagged *C. auris ERG11, MDR1,* or *CDR1* from the *S. cerevisiae PDR5* locus. The recombinant strains showed similar generation times and morphologies compared to the parental host strain ADΔΔ during culture in SD medium pH 6.8 (Figure S1, Table S4). SDS-PAGE profiles of crude membrane preparations from cells overexpressing CauErg11-6×His, CauErg11 Y132F-6×His, CauErg11 K143R-6×His, CauMdr1-6×His, and CauCdr1-6×His are shown in Figure 1a, with the corresponding Western blot in Figure 1a. As expected, the control strain Y941 [35], which overexpresses *ScERG11* at the *PDR5* locus, showed a strong band at 62 kDa in the Coomassie-stained gel, indicative of increased expression (Figure 1a, lane 2, asterisk) compared to the host strain Y1857 (ADΔΔ) (Figure 1a, lane 1, asterisk). In the corresponding Western blot, Y941 gave a sharp band (Figure 1b, lane 2), whereas the endogenous ScErg11 in Y1857 was not detected because it was not hexahistidine-tagged (Figure 1b, lane 1). The relative overexpression of CauErg11-6×His and its mutants visible on Coomassie-stained gel (Figure 1a, lane 5–7, asterisk) was evaluated by Western blot. Strains Y2767, Y2768, and Y2769 expressed their recombinant products at 0.52, 0.70, and 0.66, respectively, of the level found for ScErg11-6×His in strain Y941 (Figure 1b, lane 5–7). The expression of CauMdr1-6×His in Y2765 was not visible on Coomassie-stained SDS-PAGE at the expected size (62.06 kDa) (Figure 1a, lane 3, asterisk). Instead, the Western blot detected a broader His-tagged band ~10 kDa smaller than expected, with a relative expression level of 0.19 (Figure 1b, lane 3). The expression of CauCdr1-6×His in Y2766 gave a prominent Coomassie-stained band at the expected size of 167.49 kDa (Figure 1a, lane 4, asterisk), and the Western blot detected a band at this size with a relative expression level 0.26 compared to ScErg11-6×His in Y941 (Figure 1b, lane 4).

### 3.2. Mass Spectrometry of Tryptic Fragments Confirms Identity of Heterologously Expressed Proteins

Coomassie-stained protein bands that migrated on an 8% SDS-PAGE gel at the locations detected by Western blot analysis were excised, proteolyzed using trypsin, and the resultant peptide fragments analyzed by MS-MS spectrometry (Figure S2). The tryptic fragments gave sequence coverage of 58% for CauErg11-6×His and 62% for both CauErg11-6×His mutants, including detection of the Y132F but not the K143R mutation. The sample for the broad protein band at ~50 kDa from strain Y2765 was prepared by Ni-NTA affinity chromatography of DM-extracted crude membranes. It gave 40% coverage for CauMdr1-6×His, including both the N and C termini of the protein and the beginning of His-tag. The identity of CauCdr1-6×His expressed by strain Y2766 was confirmed based on 38% sequence coverage.

### 3.3. Susceptibility Profiles of Recombinant Strains Confirm Phenotypes

Susceptibility profiles of the host strain ADΔΔ and the recombinant strains were evaluated by measuring MIC_80_ values for FLC, VRC, POS, ITC, ISA, VT-1161, and the control non-azole antifungals MFG and AMB. Figure S3a and Table 2 show MIC_80_ values for ADΔΔ (Y1857), CauErg11 (Y2767) and its mutants Y132F (Y2768), and K143R (Y2769). As expected, the overexpression of *CauERG11-6×His* significantly reduced susceptibility to VRC and VT-1161, but not to AMB or MFG, consistent with the two azole drugs targeting CauErg11. The two CauErg11 mutants exhibited significantly higher MIC_80_ values for FLC and VRC in comparison to the host strain Y1857. The Y132F mutant also gave MIC_80_ values significantly higher than the host strain when tested against the tetrazole VT-1161, while the K143R mutant appeared susceptible to VT-1161 and showed MIC values comparable to the host strain. The MIC_80_ values for the strains expressing recombinant wild-type CauErg11 and its Y132F and K143R mutations were identical for the remaining antifungals tested.

Figure S3b and Table 2 show MIC_80_ values obtained for the strains overexpressing drug efflux pumps. Compared to the host strain Y1857, the expression of CauMdr1 (Y2765) or CauCdr1 (Y2766) increased FLC resistance >155-fold. VRC resistance increased >100-fold on expression of recombinant CauMdr1 and >200-fold on expression of recombinant CauCdr1. The expression of either efflux pump gave significantly higher MIC values with the midlength-tailed azole ISA, a more modest increase of 7-fold for CauMdr1 and a very strong increase of >800-fold for CauCdr1. Expression of CauCdr1 but not CauMdr1 elevated MIC_80_ levels against POS 4-fold, ITC > 47-fold, and VT-1161 > 2500-fold compared to the host strain. The control antifungals MFG and AMB gave essentially unchanged MIC_80_ values for the host and recombinant strains.

### 3.4. Nile Red Confirms Efflux Pump Activity

Nile Red was used as a substrate to confirm the efflux activity of strains overexpressing CauMdr1 (Y2765) and CauCdr1 (Y2766). The host strain ADΔΔ (Y1857) was used as negative control (Figure S4), and the previously described *S. cerevisiae* strains that overexpress *C. albicans* Mdr1A (Y525) or Cdr1B (Y570) provided positive controls [37]. Pump-specific inhibitors were used to confirm specificity.

**Figure 2:**
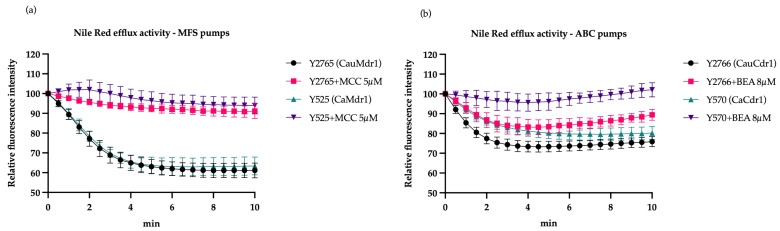
Nile Red efflux activity of recombinant fungal efflux pumps (**a**) MFS and (**b**) ABC pumps. Strains starved in the presence of 5 mM 2-DOG for 30 min were loaded with 7.5 µM Nile Red. Efflux was initiated with 20 mM glucose. As Nile Red is less fluorescent in aqueous solution than when cell bound, the fluorescence signal decreases when it has been effluxed from the cell [53]. All strains exhibited a decrease in fluorescence intensity over a period of 10 min after glucose addition. The pump-specific inhibitors not only reduced the initial rates of efflux but also the steady-state levels of Nile Red associated with the cells were maintained or returned to near-control levels. The percent mean relative fluorescence intensity for three biological replicates (*n* = 3) is shown, with each assay carried out using duplicates. The bars represent the standard deviation. MCC = MCC1189, BEA = Beauvericin.

For the MFS-pump-expressing strains Y2765 and Y525 preloaded with Nile Red, proton motive force-dependent efflux activated with glucose decreased the relative fluorescence intensity of membrane-associated Nile Red by about 35% within 3 min. After 10 min, a steady-state reduction of 40% was seen (Figure 2a). A previous study showed that compound MCC1189 is a specific inhibitor of MFS pumps [46]. The addition of 5 µM MCC1189 almost completely inhibited Nile Red efflux by strains Y525 and Y2765.

Strains Y2766 and Y570, expressing ATP-dependent ABC pumps, showed weaker glucose activation of Nile Red efflux than the MFS strains. After 3 min of glucose exposure, the membrane-associated relative fluorescence intensity was reduced by 25% for Y2766 and 20% for Y570, with similar or slightly lower steady-state levels of fluorescence intensity maintained for 10 min (Figure 2b). The addition of 8 µM Beauvericin, a known inhibitor of *C. albicans* Cdr1 and Cdr2 [54], almost completely inhibited CaCdr1-mediated efflux and inhibited, by ~50%, the rate of efflux by CauCdr1. The steady-state level of the fluorescence for strain Y2766 then gradually increased to >90% of the control level over the next 7 min. A control experiment using the known inhibitor of ABC pumps on the MFS pumps and vice versa confirmed that the activity measured in the efflux assays was pump specific (Figure S4a,b).

### 3.5. ATPase Activity

**Figure 3:**
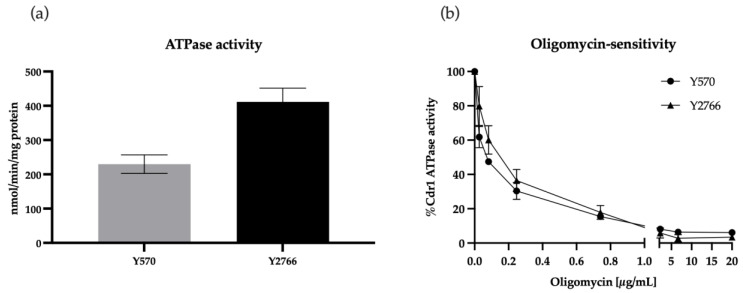
ATPase activity and OM sensitivity of strains overexpressing Cdr1. (**a**) Both strains exhibit ATPase activity. CaCdr1 (Y570) gives half the activity of CauCdr1 (Y2766). (**b**) Dose-dependent inhibition of Cdr1 ATPase activity with OM confirms the expected ATP-dependence pump function and specificity of CaCdr1 and CauCdr1. Bars represent standard deviation of 3 biological replicates (*n* = 3).

To demonstrate the ATP-dependent efflux activity of the CauCdr1-overexpressing strain, the ATPase activities of crude membranes containing recombinant CauCdr1 (Y2766) and CaCdr1B (Y570) were measured. Crude membranes from strain Y2411 were used as a negative control to correct for background ATPase activity in the Cdr1-expressing strains. Strains Y570 and Y2768 had 4-fold (Y570) or 7-fold (Y2766) higher ATPase activity, respectively, than the negative control Y2411 (57 ± 13 nmol/min/mg protein), confirming the functionality of CauCdr1. Figure 3a shows that the activity of Y2766 was almost double (411 ± 40 nmol/min/mg protein) that of Y570 (230 ± 27 nmol/min/mg protein). The crude membranes from both strains showed similar dose-dependent inhibition of ATPase activity, reaching >90% inhibition (Figure 3b) at concentrations of OM ≥ 2µg/mL. The IC_50_ values of Y2766 and Y570 Cdr1 ATPase activity for OM were 0.128 and 0.068, respectively.

### 3.6. Type II Azole-Binding Studies

The function of the recombinant CauErg11 was investigated by measuring its ability to bind the azoles POS, VRC, and ISA. CauErg11-6×His was partially purified from DM extracts of crude membranes using Ni-NTA affinity chromatography eluted with L-Histidine [55]. The amount of enzyme recovered was quantitated using its carbon monoxide binding spectra and an extinction coefficient of 91 mM^−1^ cm^−1^ [52]. The affinity-purified enzyme gave a peak at 446 nm and a shoulder peak at 420 nm, possibly indicative of a significant proportion of non-functional enzyme (Figure S5a). 

**Figure 4:**
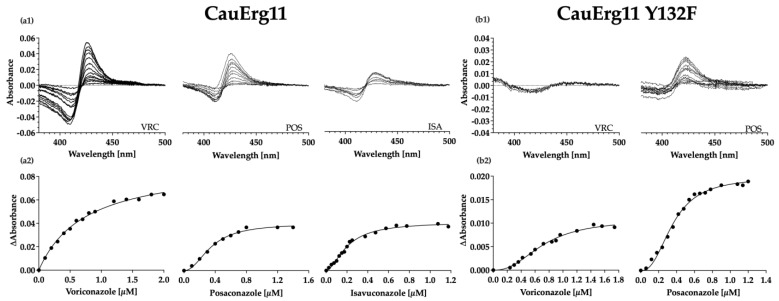
Binding studies with wild-type CauErg11-6×His and CauErg11-6×His Y132F. Type II difference spectra of 1 µM (**a1**) CauErg11 in the presence of VRC, POS or ISA, (**b1**) CauErg11 Y132F in the presence of VRC or POS. Saturation curves of 1 µM *C. auris* Erg11 proteins titrated against (**a2**) 2 µM VRC, 1.4 µM POS, and 1.2 µM ISA. (**b2**) 1.8 µM VRC and 1.2 µM POS (all diluted in DMSO).

Type II binding of the azoles (Figure 4(a1)) allowed affinity constants to be calculated from difference spectra using either the Hill equation or Michaelis–Menten binding equation (Figure 4(a2)). The equation providing best fit to the data was chosen using the Akaike’s Information Criterion (AIC). Each of the three azoles tested (VRC, ISA, and POS) gave tight 1-1 type II binding (Table 3) with *K*_d_ values < 1 µM in the presence of 1 µM enzyme.

Ni-NTA affinity purification of mutant CauErg11-6×His from strains Y2768 and Y2769 did not provide enough stable protein for binding studies. To overcome the instability of the enzyme, 40 µM FLC was added to all buffers after solubilization of crude membranes and was removed by centrifugal filtration immediately prior to type II binding studies. CO binding did not give the expected peak of 446 nm. Instead, a trough at 435 nm and a peak at 417 nm implied that CO was not bound (Figure S5b). The concentration of CauErg11 Y132F was calculated using the absolute absorbance peak maximum at 417 nm using an extinction coefficient of 117 mM^−1^ cm^−1^ [56]. Type II binding of the azoles VRC and POS gave difference spectra (Figure 4(b1)) that allowed for calculation of affinity constants (Figure 4(b2)). While the *K*_d_ values < 1 µM obtained in the presence of 1 µM enzyme indicated tight binding of both POS and VRC, comparison of the maximal trough–peak absorbance difference (Figure 4(a1,b1)) between the CauErg11 Y132F-6×His (ΔA = 0.009) and wild-type CauErg11-6×His (0.06) preparations indicated that significantly less VRC than POS (Y132F 0.02; wild type 0.04) was bound at saturation.

Poor protein stability has, thus far, precluded type II binding experiments with the K143R mutant.

## 4. Discussion

### 4.1. Functional Expression of the C. Auris Azole Target and Drug Efflux Pumps in S. cerevisiae

Functional exahistidine-tagged *C. auris* Erg11, Mdr1, and Cdr1 were overexpressed in our *S. cerevisiae* expression system [35,36,37,38,39]. Constitutive overexpression of *CauERG11-6×His*, *CauERG11 Y132F-6×His*, *CauERG11 K143R-6×His*, *CauMDR1-6×His,* and *CauCDR1-6×His* from the *S. cerevisiae PDR5* locus under the control of the *pdr1-3* transcriptional regulator gene provided a platform for phenotypic and biochemical study of these enzymes. As the host strain Y1857 has seven ABC transporters and the Pdr3 transcriptional regulator deleted, drug transport via endogenous efflux pumps is minimized, resulting in a host hypersensitive to azole drugs. In this background, the reduced susceptibility of the recombinant strains to azole drugs was primarily due to the overexpression of functional Erg11 or the efflux pumps Mdr1 or Cdr1. As *CauERG11-6×His* and its Y132F and K143R mutants complemented deletion of the native *S. cerevisiae ERG11,* these recombinant enzymes were functional despite showing significant instability on detergent extraction from crude membrane preparations.

The function of CauErg11-6×His as a drug target in recombinant strains was confirmed by its reduced susceptibility to all azoles tested compared to the host strain Y1857, plus the conferral of azole resistance at comparable or greater levels than with the previously described *S. cerevisiae* Erg11 Y140F mutation and the *C. albicans* Erg11 Y132F and K143R mutations [57,58]. In addition, type II binding of POS, VRC, and ISA was demonstrated for CauErg11-6×His*,* with all three drugs giving dissociation constants (*K*_d_) <1 indicative of tight 1-1 binding. Previous studies of overexpressed *C. albicans* and *C. parapsilosis* CYP51 gave *K*_d_ values in a similar range for the antifungal POS [36,38]. The affinity of VRC for CauErg11-6×His was found to be ~2.5-fold lower (K_d_ = 0.74) than for CaErg11-6×His (K_d_ = 0.29) [38]. The exact reason for this is not known but is likely to be due to subtle differences in the conformation of the active site. CauErg11-6×His Y132F and K132R did not give stable protein on detergent extraction; therefore, 40 µM FLC was used to stabilize the Y132F mutant enzyme during affinity purification. This sufficiently stabilized CauErg11-6×His Y132F to enable type II binding studies despite a lack of CO binding. Type II binding measurements indicated tight 1-1 binding with a stronger affinity for POS (*K*_d_ = 0.36) than for VRC (K_d_ = 0.7), in both the wild-type and Y132F mutant enzyme. While the binding of POS to CauErg11 declined by 2-fold, VRC binding decreased 9-fold between the wild-type and mutant enzymes, suggesting that VRC is less able than POS to occupy the CauErg11 Y132F-6×His active site (Figure 4(b1)). The comparable experiment was not carried out with the K143R mutant due to even lower protein stability. Type II binding studies as well as the addition of FLC as an enzyme stabilizer are not ideal and it may be preferable to measure inhibitor binding in crude membrane preparations using the BOMCC assay [59]. However, this approach may require the introduction of a cognate NADPH-cytochrome P450 reductase.

CauMdr1-6×His and CauCdr1-6×His function were confirmed by measuring glucose-induced efflux pump activity using Nile Red as a substrate. The activity of CauMdr1-6×His was inhibited by 5 µM MCC1189, a compound shown to specifically inhibit *C. albicans* Mdr1 [46]. As it is a demonstrated substrate for CaCdr1 [46], MCC1189 also appeared to be a weak inhibitor of Nile Red efflux by CauCdr1. CauCdr1, but not CauMdr1, was inhibited by 8 µM Beauvericin. This drug was previously shown to inhibit *C. albicans* Cdr1 and Cdr2 [54]. The biochemical function of CauCdr1 was confirmed by determining its ATPase activity and OM sensitivity. The PDR subfamily of ABC transporters is OM sensitive [60], and previous studies have shown that OM inhibits *C. albicans* Cdr1 ATPase activity [37,61]. The CauCdr1-6×His ATPase was twice as active as its *C. albicans* counterpart and showed comparable dose-dependent OM inhibition.

In SDS-PAGE, CauMdr1-6×His appeared as smear migrating ~10 kDa smaller than expected. Gel shifting is common for membrane proteins [62], with some integral membrane proteins shown to migrate faster at relative migrations of 0.82 × MW during SDS-PAGE [63]. This property appears applicable to CauMdr1-6×His*,* which has a molecular weight of 62.05 kDa and displayed a relative migration equivalent to ~51 kDa (Figure 1b). Mass spectrometry of tryptic fragments clearly identified full-length CauMdr1 by providing a primary sequence coverage of 40%, including both N and C termini of the protein and the beginning of the His-tag. The modest overall level of sequence coverage may be because the central portion of the protein is highly hydrophobic and contains few tryptic cleavage sites. A similar problem may be inherent to the mass spectrometry analysis of the recombinant CauCdr1, which gave 38% sequence coverage.

### 4.2. Azole Susceptibility Wild-Type CauErg11 and the Y132F and K143R Mutants

Susceptibility assays demonstrated that the CauErg11 Y132F and K143R mutations conferred resistance to short-tailed triazoles FLC and VRC but not to the long-tailed triazoles POS and ITC, or the mid-tailed triazole ISA. Furthermore, the Y132F mutation conferred several-fold stronger resistance to FLC and VRC than the K143R mutation. The Y132F mutation also conferred weak resistance to the midlength-tailed tetrazole VT-1161. In contrast, the K143R mutation gave slightly increased susceptibility to VT-1161.

In *C. albicans,* Erg11 Y132F and K143R are known contributors to azole resistance [64]. The CaErg11 Y132F increased the MIC of FLC ≥4-fold and the K143R conferred even higher resistance, with a 16-fold increase [64]. Both mutations also increased resistance to VRC and to ITC (by <2-fold) but did not increase resistance to POS. Crystal structures of ScErg11 show that Y140 forms a water-mediated hydrogen bond with the tertiary alcohol of FLC and VRC (PDB #: 4WMZ and 5HS1, respectively). The homologous CaErg11 Y132 residue (PDB #: 5V5Z) is suitably situated within the catalytic site to enable formation of a similar water-mediated hydrogen bond network with the tertiary alcohol of FLC, VRC, and VT-1161. In both CaErg11 and CauErg11, the Y132F mutation, as expected, increased the MIC_80_ isignificantly compared with CauErg11 for FLC (60-fold) and VRC (9-fold), and weakly for VT-1161 (2.6-fold), but not for ITC or POS. The binding of ISA, which contains a tertiary alcohol group, was not affected by the CauErg11 Y132F mutation. This is presumably because of compensatory interactions with the active site of the enzyme, possibly due to the 2,5-difluorphenyl group of ISA, its stereochemistry around the tertiary alcohol, and/or due to interactions of its butan-2-yl-1,3-thiazol-4-ylbenzonitrile tail with the entry channel of Erg11. Interestingly, the homologous *S. cerevisiae* Erg11 Y140F mutation does not confer resistance to ISA.

In the CaErg11 crystal structure (PDB #: 5V5Z) and in a homology model of CauErg11, K143 lies in a position proximal to the heme iron, forming an ionic bond with the heme propionate that is hydrogen bonded with Y132. The K143R mutation is, therefore, predicted to indirectly affect the binding of substrates interacting with Y132 and affected by the Y132F mutation. While the CauErg11 K143R mutant expressed in *S. cerevisiae* conferred resistance to FLC (16-fold MIC_80_ increase versus wild-type CauErg11) and VRC (2.3-fold), the level of resistance was lower than for the CauErg11 Y132F mutant, presumably due to the indirect effect of the K143 mutation on Y132. As expected from the analysis of the Y132F mutant, the CauErg11 K143R mutant did not confer resistance to ISA, ITC, and POS. It also failed to confer resistance to VT-1161. The reason for this difference is not clear but the indirect action of the K143R mutation may have been insufficient to affect the key hydrogen bond network involving CauErg11 Y132. The K143R mutation is also predicted to affect the catalytic efficiency of the enzyme [58]. As the strains expressing CauErg11, CauErg11 Y132F, and CauERG11 K143R all grew at comparable rates (Figure S1, Table S4) and gave identical growth yields at 30 °C in SD medium at pH 6.8, any impact on this intermolecular interaction did not appear critical for these strains that require the recombinant enzyme for growth.

### 4.3. Susceptibility Efflux Pumps Mdr1 and Cdr1

The overexpression of CauMdr1 confers strong resistance to FLC and VRC, and weaker resistance to VT-1161, but not to ITC or POS (Figure S3b, Table 2). Overexpressed Cdr1-6×His conferred strong resistance to all azoles tested, i.e., FLC, VRC, VT-1161, ISA, ITC, and POS (Figure S3b, Table 2). The overexpressed Mdr1 and Cdr1 efflux pumps in *C. albicans* and *C. auris* contribute in similar fashions to intermediate and high-level azole resistance, respectively. Our data suggest that CauMdr1-overexpressing strains will mainly contribute to FLC and VRC resistance, whereas the overexpression of CauCdr1 is the major driver of pan-azole resistance. Constitutive high-level expression of these efflux pumps conferred by GOF mutations in the Mrr1 and Tac1b transcription factors, mimicked in our *S. cerevisiae* model by the Pdr1-3 transcription factor acting on the *PDR5* locus, can be expected to dominate the azole resistance in *C. auris* Clades I and IV conferred by overexpression of CauErg11 and by the mutations Y132F and K143R. Our successful use of the *pdr1-3* GOF transcriptional regulator gene as a surrogate is much simpler than, for example, engineering *S. cerevisiae* strains in which the *ScMRR1* or *ScPDR1* loci are replaced with their wild-type and mutant *CauMRR1* or *CauTAC1* counterparts, as well as requiring modification of the *PDR5* promoter in anticipation that functional heterologous transcription complexes will be formed. In contrast, reversion of the *pdr1-3* gene to *PDR1* in the *S. cerevisiae CauMDR1* and *CauCDR1* recombinant strains might enable mimicry of wild-type CauMdr1 and CauCdr1 expression and function.

### 4.4. Conclusions

A *S. cerevisiae-*based expression system with background drug efflux pumps deleted was used to functionally overexpress the recombinant azole target CauErg11, its Y132F and K143R mutants, the MFS drug efflux pump CauMdr1, and the ABC transporter CauCdr1. This approach, in a host system hypersensitive to azole drugs, enabled characterization of the azole drug target CauErg11, an essential contributor to sterol biosynthesis, the main target-based mutations (CauErg11 Y132F and K143R), plus the CauMdr1- and CauCdr1-dependent drug efflux pumps that contribute to azole resistance in *C. auris*. This platform extends our previous studies with key molecules from other fungal pathogens expressed in *S. cerevisiae* [36,38,46,65,66,67]. It provides important tools for phenotypic and biochemical screens of these key targets affecting azole susceptibility and/or resistance. The platform has its limitations because the proteins are overexpressed in high levels, which may not fully reflect their natural occurrence. While this model cannot replace the testing of lead compounds with *C. auris* clinical isolates, it creates an opportunity for the structure-based discovery needed to identify novel azole antifungals not susceptible to target-based resistance as well as screens for inhibitors of the MFS and ABC transporter-based drug efflux mechanisms that contribute high-level azole resistance.

## Figures and Tables

**Table 1:** Common resistance mechanism in *C. auris*.

Antifungal Class	Drug Target	Resistance Mechanism in *C. auris*	Clade	References
**Azoles**	Erg11	**Erg11 point mutation**		[7,12,15,16,17,18]
		F126L	III	
		Y132F	I, IV, V	
		K143R	I, IV	
		**Erg11 copy number variants**	I, III	[15,19]
		**Efflux pump gene overexpression** *MDR1* and *CDR1*	I, III, IV	[20,21]
		**Mutation in Tac1b**		[22,23,24]
		A640V	I	
		A657V	I	
		F862_N866del	IV	
		**Mutation in Mrr1**		
		N647T	III	
**Echinocandins**	β-1,3-glucan synthase	**Mutation in Fks1 hot spot 1**	I, III, IV	[17,25,26,27,28]
		S639F/P/Y		
		F635Y/L		
		**Mutation in Fks1 hot spot 2**	I, III, IV	
		R1354S		
**Polyenes**	Ergosterol	**Primary mechanism unknown** *ERG6* mutations	I, III, IV	[29,30]

**Table 2:** Susceptibilities of the host and recombinant strains developed in this study.

	Average MIC_80_ in µM/mg·L^−1^							
Strain	FLC	VRC	POS	ITC	ISA	VT-1161	MFG	AMB
ADΔΔ (host)	2.58/0.8	0.08/0.03	0.22/0.15	0.21/0.15	0.015/0.007	0.018/0.009	0.23/0.29	3.31/3.06
CauErg11	3.90/1.2 (1.5)	0.22/0.08 (2.8)	0.33/0.23 (1.5)	0.21/0.15 (1)	0.020/0.009 (1.3)	0.05/0.03 (2.8)	0.50/0.64 (2.2)	5/4.62 (1.5)
CauErg11 Y132F	250/76.6 (97)	2/0.7 (25)	0.20/0.14 (0.9)	0.20/0.14 (0.95)	0.015/0.007 (1)	0.125/0.07 (7)	0.34/0.43 (1.5)	3.12/2.88 (0.9)
CauErg11 K143R	56.5/17.3 (22)	0.50/0.17 (6.25)	0.18/0.12 (0.8)	0.125/0.09 (0.6)	0.020/0.009 (1.3)	0.03/0.02 (1.7)	0.34/0.43 (1.5)	3.75/3.47 (1.1)
CauMdr1	>400/>122 (>155)	10.31/3.6 (129)	0.20/0.14 (0.9)	0.23/0.16 (1.1)	0.10/0.04 (6.7)	0.068/0.04 (3.8)	0.24/0.3 (1)	2.58/2.38 (0.8)
CauCdr1	>400/>122 (>155)	20.63/7.2 (258)	1.08/0.76 (5)	>10/>7.06 (>47)	12.5/5.5 (833)	>50/>26.4 (>2777)	0.23/0.29 (1)	1.90/1.76 (0.6)

>MIC_80_ higher than shown. Values in brackets indicate fold change in MIC_80_ compared to ADΔΔ.

**Table 3:** Type II binding of azoles to CauErg11-6×His and CauErg11 Y132F-6×His.

Strain Designation	Azole	ΔAmax	Best Fit Equation	*K*_d_ (µM)
CauErg11	Voriconazole	0.09	Michaelis-Menten	0.74 (±0.09)
CauErg11	Posaconazole	0.04	Hill	0.34 (±0.04)
CauErg11	Isavuconazole	0.04	Hill	0.20 (±0.02)
CauErg11 Y132F	Voriconazole	0.01	Hill	0.7
CauErg11 Y132F	Posaconazole	0.02	Hill	0.36

Values in brackets indicate standard error.

## Data Availability

Not applicable.

## Supplementary Materials

The following supporting information can be downloaded at: https://www.mdpi.com/article/10.3390/jof9020168/s1, Table S1: List of strains used in this study, Table S2: Oligonucleotides used in this study, Table S3: Nucleotide sequences of the ORFs of interest, Table S4: Generation times of strains used in this study, Figure S1: Generation times of strains used in this study, Figure S2: Mass spectrometry analysis of tryptic fragments of protein bands, Figure S3: Drug susceptibilities of strains used in this study, Figure S4: Nile Red assays with control compounds, Figure S5: Carbon monoxide binding by wild-type CauErg11 and CauErg11 Y132F.
