# Supplementary material for: Functional Expression of Recombinant Candida auris Proteins in Saccharomyces cerevisiae Enables Azole Susceptibility Evaluation and Drug Discovery
Source: J Fungi (Basel). 2023 Jan 27;9(2):168. doi: 10.3390/jof9020168 (PMC9960696; doi:10.3390/jof9020168)
Supplement: Supplementary file 1 [file jof-09-00168-s001.zip › jof-2145332-supplementary.docx]

Functional expression of recombinant *Candida auris* proteins in *Saccharomyces cerevisiae* enables azole susceptibility evaluation and drug discovery

Stephanie Toepfer, Michaela Lackner, Mikhail V. Keniya, and Brian C. Monk

**Supplementary Table S1.**

**Table S1**. List of strains used in this study.

| **Strain** | **Genotype** | **Description** | **Source** |
| --- | --- | --- | --- |
| AD | *MAT*α *pdr1-3 ura3 his1 Δyor1::hisG Δsnq2::hisG Δpdr10::hisG Δpdr11::hisG Δycf1::hisG Δpdr3::hisG Δpdr5::hisG Δpdr15::hisG* | AD1-8u- | [67] |
| ADΔ | *MAT*α *pdr1-3 his1 Δyor1::hisG Δsnq2::hisG*  *Δpdr10::hisG Δpdr11::hisG Δycf1::hisG Δpdr3::hisG Δpdr5::hisG Δpdr15::hisG, Δura3* | AD1-8u- Δ*URA3* | [37] |
| Y1857 | ADΔ, *Δhis1::dpl200* | ADΔΔ | [66] |
| Y2411 | ADΔΔ, *Δpdr5::*pABC3 | ADΔΔ with empty pABC3 (*URA3*) at *PDR5* locus | [48] |
| Y941 | ADΔ, *Δpdr5::ScCYP51-6×His URA3* | *S. cerevisiae* Erg11 overexpressed | [37] |
| Y525 | AD *Δpdr5::CaMDR1A* | *C. albicans* Mdr1A overexpressed | [37] |
| Y570 | AD *Δpdr5::CaCDR1B* | *C. albicans* Cdr1b overexpressed | [37] |
| Y2765 | ADΔΔ *Δpdr5::CauMDR1-6×His LoxP-HIS1-LoxP* | *C. auris* Mdr1 overexpressed | This study |
| Y2766 | ADΔΔ *Δpdr5::CauCDR1-6×His LoxP-HIS1-LoxP* | *C. auris* Cdr1 overexpressed | This study |
| Y2767 | ADΔΔ *Δpdr5::CauERG11-6×His LoxP-HIS1-LoxP*  *Δerg11::LoxM2-URA3-LoxM2* | *C. auris* Erg11 overexpressed  & *S. cerevisiae ERG11* deleted | This study |
| Y2768 | ADΔΔ *Δpdr5::CauERG11 Y132F-6×His* | Y2767 with Erg11 Y132F mutation | This study |
| Y2769 | ADΔΔ *Δpdr5::CauERG11 K143R-6×His* | Y2767 with Erg11 K143R mutation | This study |

**Supplementary Table S2.**

**Table S2**. Oligonucleotides used in this study.

| **Primer name** | **Sequence (5’ – 3’)** | **Amplification/creation of** |
| --- | --- | --- |
| PDR5Fv3 | TCGCATTCTGCGCCTTCGAGCAC | **PDR5::GOI cassette from gDNA of selected strains** |
| PDRF_186DS_R | TTCGGACATTGAACTTTGATTTATC |  |
| CauERG11US120_F | CTAAAAGAAACCCGTACACCATCG | **Erg11, Mdr1, Cdr1 from gDNA of B11220** |
| CauERG11DS101_R | CTGGCCTGAGTCACCACTGTATGC |  |
| CauMDR1US125_F | ATGCCATTTTTGCTCTCATTACTC |  |
| CauMDR1DS1755_R | CGTATTTGTTCTACACAAGAAAAC |  |
| CauCDR1US119_F | CGTAGCTGGGGCTGCTTCTACCTC |  |
| CauCDRDS4645_R | CTTAACTGACTCAGGGTAATTATAC |  |
| FU_pacI_ERG11ORF0_F | GCTCGTTCGAAAGACTTAATTAAAAAATGGCCTTGAAGGACTGCATC | **CauErg11-6×His transformation cassette** |
| FU_CauERG11Not1-6xHis_R | TTAATGATGATGGTGATGATGGCGGCCGCCGTAAACACAAG |  |
| FU_CauERG11_Y132F_f | CCAGTTTTCGGGAAAGGTGTCATTTTCGACTGTCCCAACTCGAGGTTGATG | **CauErg11 Y132F-6×His transformation cassette from CauERG11-6×His** |
| FU_CauERG11_Y132F_r | CATCAACCTCGAGTTGGGACAGTCGAAAATGACACCTTTCCCGAAAACTGG |  |
| FU_CauERG11_K143R_f | CCAACTCGAGGTTGATGGAGCAGAGGAAATTTGCTAAGACTGCCTTGACG | **CauErg11 K143R-6×His transformation cassette from CauErg11-6×His** |
| FU_CauERG11_K143R_r | CGTCAAGGCAGTCTTAGCAAATTTCCTCTGCTCCATCAACCTCGAGTTGG |  |
| FU_pacI_MDR1ORF0_F | GCTCGTTCGAAAGACTTAATTAAAAAATGTTCCTCTATAAATTCGTC | **CauMdr1-6×His transformation cassette** |
| FU_CauMDR1Not1-6xHis_R | TTAATGATGATGGTGATGATGGCGGCCGCCGGCACCTGCTC |  |
| FU_pacI_CDR1ORF0_F | GCTCGTTCGAAAGACTTAATTAAAAAATGTCCGAGAAACCTTTTGTC | **CauCdr1-6×His transformation cassette** |
| FU_CauCDR1Not1-6xHis_R | TTAATGATGATGGTGATGATGGCGGCCGCCGTGGTTTTTAGACTTGGAAG |  |

F/f, R/r stand for forward and reverse, respectively.

**Supplementary Table S3.** Nucleotide sequences of the ORFs of interest.

Sequence of CauErg11-6×His ORF

ATGGCCTTGAAGGACTGCATCGTCGATGTTGTCGACCGATTTTCGGCGTTGCCCGTGCCCGTGAAGCTCGCGGTGCTTATTTTGGTGCCCATCGTCTACAACCTTGTATGGCAGTTTGTCTACTCTCTCAGAAAAGACAGAGCTCCCTTAGTGTTTCACTGGGTGCCATGGGTGGGCTCTGCTGTTGTTTACGGAATGCAGCCATATCAATTTTTCGAGCTGTGCAGAGAGAAATACGGCGATGTGTTTGCTTTCGTGATGTTAGGAAAAGTTATGACGGTTTACTTGGGACCTAAGGGACACGAGTTTGTATTGAACGCTAAGCTTGCGGATGTTTCTGCCGAGGCTGCTTATTCCCACTTGACCACTCCAGTTTTCGGGAAAGGTGTCATTTACGACTGTCCCAACTCGAGGTTGATGGAGCAGAAGAAATTTGCTAAGACTGCCTTGACGAAAGAAGCTTTCCAGAGGTACGTGCCCAGAATCCAAGAGGAAGTTTTGGACTACTTCAAAGCTTGCTCCCAATTCAAAATGAACGAACGTAACAACGGCGTGGCCAATGTGATGAAGACTCAGCCTGAGATGACCATCTTAACTGCTTCCAAGTCGTTGATGGGTGATGACATGAGAGCCAGATTTGATGCCTCCTTCGCCAAATTGTACTCCGACTTGGATAAAGGTTTCACACCCATCAACTTCGTCTTCCCCCACTTGCCCTTGCCTGCCTACTGGAAGAGAGACGCTGCTCAGCAGAAGATCTCTGCTACCTACATGTCCTTGATTAACGAGAGAAGAAAGACCGGTGACATCGTTCCTGACAGGGACTTGATCGACTCGCTAATGACAAACTCGACGTATAAAGACGGCGTGAAGATGACCGATCAGGAGGTTGCCAACTTGTTGATTGGTGTTTTGATGGGAGGTCAGCACACTTCAGCTTCCACGTCTGCCTGGTTTTTGTTGCACTTAGCTGAGCAACCAAAGTTGCAAGAGGAGCTTTACAATGAGGTCCTTTCAGTTTTGGCTGAAAAGGGCGGCAGCTTAAAGGATTTGGCTTACGACGACTTACAGAAGATGCCTTTGATCAACCAGACCATCAAGGAGACGTTGCGTTTGCACATGCCATTGCACTCCATTTTCAGAAAAGTCATGAACCCACTCGTGGTTCCAAACACCAAGTATGTTGTGCCTAAGGGTCACTACGTGATGGTCTCCCCAGGTTACGCCCAGACCAACGAGAAGTGGTTCCCCAGGGCCAACGAGTTCGACCCACACAGATGGGACGAGGAAACCTCCAGCAATATAGACACCGATGCTGTCGACTACGGGTTTGGTAAAGTCACCAAGGGTGTTTCTTCGCCATACTTGCCTTTTGGCGGTGGTAGACACCGTTGCATTGGTGAGCAGTTTGCCTACGTGCAATTGGGTACCATCTTAGCTACCTATGTGTACAACATCAAGTGGAGGTTCAAGAAGGATGGATCGCTTCCACCAGTCGACTACCAGTCGATGGTGACGCTACCAATGGAGCCAGCAGAAATCGAGTGGGAGAAAAGAGAGACTTGTGTTTACGGCggccgccatcatcaccatcatcatTAA

Sequence of CauErg11 Y132F-6×His ORF

ATGGCCTTGAAGGACTGCATCGTCGATGTTGTCGACCGATTTTCGGCGTTGCCCGTGCCCGTGAAGCTCGCGGTGCTTATTTTGGTGCCCATCGTCTACAACCTTGTATGGCAGTTTGTCTACTCTCTCAGAAAAGACAGAGCTCCCTTAGTGTTTCACTGGGTGCCATGGGTGGGCTCTGCTGTTGTTTACGGAATGCAGCCATATCAATTTTTCGAGCTGTGCAGAGAGAAATACGGCGATGTGTTTGCTTTCGTGATGTTAGGAAAAGTTATGACGGTTTACTTGGGACCTAAGGGACACGAGTTTGTATTGAACGCTAAGCTTGCGGATGTTTCTGCCGAGGCTGCTTATTCCCACTTGACCACTCCAGTTTTCGGGAAAGGTGTCATT**TTC**GACTGTCCCAACTCGAGGTTGATGGAGCAGAAGAAATTTGCTAAGACTGCCTTGACGAAAGAAGCTTTCCAGAGGTACGTGCCCAGAATCCAAGAGGAAGTTTTGGACTACTTCAAAGCTTGCTCCCAATTCAAAATGAACGAACGTAACAACGGCGTGGCCAATGTGATGAAGACTCAGCCTGAGATGACCATCTTAACTGCTTCCAAGTCGTTGATGGGTGATGACATGAGAGCCAGATTTGATGCCTCCTTCGCCAAATTGTACTCCGACTTGGATAAAGGTTTCACACCCATCAACTTCGTCTTCCCCCACTTGCCCTTGCCTGCCTACTGGAAGAGAGACGCTGCTCAGCAGAAGATCTCTGCTACCTACATGTCCTTGATTAACGAGAGAAGAAAGACCGGTGACATCGTTCCTGACAGGGACTTGATCGACTCGCTAATGACAAACTCGACGTATAAAGACGGCGTGAAGATGACCGATCAGGAGGTTGCCAACTTGTTGATTGGTGTTTTGATGGGAGGTCAGCACACTTCAGCTTCCACGTCTGCCTGGTTTTTGTTGCACTTAGCTGAGCAACCAAAGTTGCAAGAGGAGCTTTACAATGAGGTCCTTTCAGTTTTGGCTGAAAAGGGCGGCAGCTTAAAGGATTTGGCTTACGACGACTTACAGAAGATGCCTTTGATCAACCAGACCATCAAGGAGACGTTGCGTTTGCACATGCCATTGCACTCCATTTTCAGAAAAGTCATGAACCCACTCGTGGTTCCAAACACCAAGTATGTTGTGCCTAAGGGTCACTACGTGATGGTCTCCCCAGGTTACGCCCAGACCAACGAGAAGTGGTTCCCCAGGGCCAACGAGTTCGACCCACACAGATGGGACGAGGAAACCTCCAGCAATATAGACACCGATGCTGTCGACTACGGGTTTGGTAAAGTCACCAAGGGTGTTTCTTCGCCATACTTGCCTTTTGGCGGTGGTAGACACCGTTGCATTGGTGAGCAGTTTGCCTACGTGCAATTGGGTACCATCTTAGCTACCTATGTGTACAACATCAAGTGGAGGTTCAAGAAGGATGGATCGCTTCCACCAGTCGACTACCAGTCGATGGTGACGCTACCAATGGAGCCAGCAGAAATCGAGTGGGAGAAAAGAGAGACTTGTGTTTACGGCggccgccatcatcaccatcatcatTAA

Sequence of CauErg11 K143R-6×His ORF

ATGGCCTTGAAGGACTGCATCGTCGATGTTGTCGACCGATTTTCGGCGTTGCCCGTGCCCGTGAAGCTCGCGGTGCTTATTTTGGTGCCCATCGTCTACAACCTTGTATGGCAGTTTGTCTACTCTCTCAGAAAAGACAGAGCTCCCTTAGTGTTTCACTGGGTGCCATGGGTGGGCTCTGCTGTTGTTTACGGAATGCAGCCATATCAATTTTTCGAGCTGTGCAGAGAGAAATACGGCGATGTGTTTGCTTTCGTGATGTTAGGAAAAGTTATGACGGTTTACTTGGGACCTAAGGGACACGAGTTTGTATTGAACGCTAAGCTTGCGGATGTTTCTGCCGAGGCTGCTTATTCCCACTTGACCACTCCAGTTTTCGGGAAAGGTGTCATTTACGACTGTCCCAACTCGAGGTTGATGGAGCAG**AGG**AAATTTGCTAAGACTGCCTTGACGAAAGAAGCTTTCCAGAGGTACGTGCCCAGAATCCAAGAGGAAGTTTTGGACTACTTCAAAGCTTGCTCCCAATTCAAAATGAACGAACGTAACAACGGCGTGGCCAATGTGATGAAGACTCAGCCTGAGATGACCATCTTAACTGCTTCCAAGTCGTTGATGGGTGATGACATGAGAGCCAGATTTGATGCCTCCTTCGCCAAATTGTACTCCGACTTGGATAAAGGTTTCACACCCATCAACTTCGTCTTCCCCCACTTGCCCTTGCCTGCCTACTGGAAGAGAGACGCTGCTCAGCAGAAGATCTCTGCTACCTACATGTCCTTGATTAACGAGAGAAGAAAGACCGGTGACATCGTTCCTGACAGGGACTTGATCGACTCGCTAATGACAAACTCGACGTATAAAGACGGCGTGAAGATGACCGATCAGGAGGTTGCCAACTTGTTGATTGGTGTTTTGATGGGAGGTCAGCACACTTCAGCTTCCACGTCTGCCTGGTTTTTGTTGCACTTAGCTGAGCAACCAAAGTTGCAAGAGGAGCTTTACAATGAGGTCCTTTCAGTTTTGGCTGAAAAGGGCGGCAGCTTAAAGGATTTGGCTTACGACGACTTACAGAAGATGCCTTTGATCAACCAGACCATCAAGGAGACGTTGCGTTTGCACATGCCATTGCACTCCATTTTCAGAAAAGTCATGAACCCACTCGTGGTTCCAAACACCAAGTATGTTGTGCCTAAGGGTCACTACGTGATGGTCTCCCCAGGTTACGCCCAGACCAACGAGAAGTGGTTCCCCAGGGCCAACGAGTTCGACCCACACAGATGGGACGAGGAAACCTCCAGCAATATAGACACCGATGCTGTCGACTACGGGTTTGGTAAAGTCACCAAGGGTGTTTCTTCGCCATACTTGCCTTTTGGCGGTGGTAGACACCGTTGCATTGGTGAGCAGTTTGCCTACGTGCAATTGGGTACCATCTTAGCTACCTATGTGTACAACATCAAGTGGAGGTTCAAGAAGGATGGATCGCTTCCACCAGTCGACTACCAGTCGATGGTGACGCTACCAATGGAGCCAGCAGAAATCGAGTGGGAGAAAAGAGAGACTTGTGTTTACGGCggccgccatcatcaccatcatcatTAA

Sequence of CauMdr1-6×His ORF

ATGTTCCTCTATAAATTCGTCAGAGAGAGCTTCTTCGGCAGGTCTCTATATCACTTATCTGGACGCAAAGTGTTCACATACCCCGAGGAATCACCCGACTATGTGATTCCCGCAAAGTACTTGGGCAAAGACGAAGCAGGCATTGAATCGGATGTTAAGGAGAAAGCTGGCGCTTCAGACACCCCCGTTGATCTGGACTCTTCGTCCCAGTCGACCAAGACCAACCACATTCTTGTGGACTGGGAGGGAGAAGACGATCCAGAAAATCCATACAATTGGCCATTGAAATACAAGATTATCTTCATTGCCCAGATCATGATTTTGACTGCATTTGTGTATATGGCTTCTGCCATTTATACCCCAGGTATTGAGGAGATTATGAAAGATATGGGCGTGGGCCAGGTGGTGGCGACACTCCCCTTGACGCTTTTTGTGTTCGGATACGGTATCGGGCCCATGGTGTTTTCGCCGCTTTCGGAAAATGCCAGGTTTGGCAGAACGTCCATCTACATCATTACCTTATTTATCTTCTTCATCTTGCAGATCCCCACGGCTCTCGTGGACAACATCGCTGGCTTGTGTATTTTGCGTTTCATCGCTGGCTTCTTTGCCAGCCCTTGTTTGGCTACCGGTGGTGCTTCTGTGGGTGACGTGATCAATTTACCTTACATTCCAGTGGGTATCTCATTCTGGTCCATCGCTGCCGTGTGTGCTCCTTCTTCGGGCCCCTTGTTTGGTGCTATCTTGTCTGTCAAGGCTGACTACCACTGGACCTTCTGGTTTGTGTGTATCACTTCTGGTGCCTCCTTTGTCGTTCTTGGTTGGATGCTTCCTGAATCTTACTCCAAAACCATTTTGTACAGAAAGGCTGAGAGACTCAGAGCTCTCACGGGTAACCAGGATATTGTCAGTGAGGGTCACCTTGAGAATGCTAAGTTCTCCACTCATGAGATGCTTGTCGAGACACTCTGGCGTCCATTTGAGGTGATTATCTTCGAGCCTGTGGTGTTGCTTATAAATATCTACATTGGCTTGGTGTACTCTGTAATGTACTTGTGGTTTGAGGCTTTCCCCATTGTGTTCGTTCAGGTCAAGGGCTTCACTCTCATCGAGATGGGTGTCGCTTACATGTGTATCTTGGTCGGTATTTTGATAGCTGCTGCATTCTACATCCCTACCATATACCATCAATTCACCAAGAAGATGCTCAGCAACCAGGAAGTCGTTCCTGAAGTCTTCATTCCCATGGCTATTGTTGGAAGTATCATCATGCCTATTGGAATCTTCATCTTTGGCTGGACGGCTGCTGAGGACCTCCACTGGATTGGTCCACTTATTGGAGCTGCAGTCTTTGCCGCTGGAGCTTTCTTGGTTTTCCAGACCTTGTTTAACTACCTCAGTATGTCATTCTGGAGATATTTGGCTTCTGTGTTTGCAGGCAATGACTTATTCAGAAGTATGATGGCGGGTGCCTTCCCACTTTTTGGCAAGCCTTTGTTCTTGAACTTGAGAACCAATAGATTCGCTGTTGGCTGGGGCTCGTCTCTCTTGGGCTTTATTTGTGTGGGCATGATTGCAATCCCAGTATTATTCTACCTCAACGGCCCTAAGTTAAGAGCTAGATCCAAGTACGCTGGAGCAGGTGCCGGCggccgccatcatcaccatcatcatTAA

Sequence of CauCdr1-6×His ORF

ATGTCCGAGAAACCTTTTGTCGACGCTCCTCCACCCGAGGATGGCGTTGCTCACCAAGTGCTGCCCCATGACAACGGCAGTCTCAGTGAGGAGGCCAATTCCATCAATGAGTATACTGGTTTTGGTGCTCATCAGGAAGGTGAAATTAGAGAGTTGGCCAGAACCTTCACCAACATGTCCCATGACTCCGGCCACGACTTATCCAAAACAAACACATCCCAGGATTTGCTCAAGTACTTGTCCCACATGTCTGAGGTGCCTGGCGTAGAGCCCTTTGACCCAGAGCAGATCAGCGAGCAGTTGAACCCAGACTCGCCCAACTTCAATGCGAAGTTTTGGGTGAAAAATATGCGTAAGTTGTTCGATTCCAACCCTGACTACTATAAGCCTTCAAAGTTGGGACTTGCGTACCGTAATTTGAGAGCCTACGGTGTGGCTGCAGACTCAGACTACCAGCCAACCGTCAGTAACGGGTTGTGGAAAATGGCGGTGGATTACTGGCACGATATGAGAAAAATCGACGAGAGCCGTTGTTTTGACATCTTAAAGACCATGGACGGGTACTTCAAGCCCGGTGAAGTCACGGTGGTGTTGGGTCGTCCTGGTTCTGGTTGTTCCACTTTGCTCAAGACGATTGCATGCAACACGTACGGTTTTCACATTGGCGAAGAATCTCAAATCTCCTATGACGGCATGACTCCAGATGAGATCCACAAACACCACCGTGGTGACGTTGTGTACTCTGCTGAGACCGATGTCCACTTTCCACACTTGAGCGTGGGGGACACTTTGGAATTCGCTGCCAAATTGAGAACGCCTCAAAACAGGGGTGAAGTTTCCAGATTGGAACACGCCAAACACATGGCCTCCGTCACCATGGCCACTTACGGTTTGTCCCACACTAGAAACACTCCTGTGGGTAACGACTTTGTCCGTGGTGTTTCTGGTGGTGAGCGTAAGCGTGTGTCTATTGCCGAGGTTTCCTTGTCTGGTGCGAACATTCAGTGCTGGGATAACGCCACGAGAGGTTTGGATGCCGCCACCGCTTTGGAGTTTATTCGTGCTTTAAAGACCTCCGCGGCCATCTTGGATGCCACCCCTTTGATTGCCATCTACCAGTGTTCCCAGGATGCTTATGACTTGTTTGACAATGTCATTGTCTTGTACGAAGGATATCAGATTTTCTTTGGTAAGGCCAGCGAAGCCAAGCAATTCTTCTTGGATATGGGTTACGAGTGCCCTCAGCGTCAAACCACCGCCGATTTCTTGACTTCCTTGACTAATCCAGAGGAGCGTGTTGTTAAGCCTGGGTTCGAAAACAAGGTTCCTCGCACTGCGAAAGAATTCTCCGATTATTGGAGAAACTCATCAAACTACAAGGTTCTCACTGCAGGCATAGACAAGTACCTTGCCGAGGTGGCCGACGGAAGCCAGAGAGAAGCTTACCGTGCATCCCACGTTGCTAAACAGTCCGACCACACTCGTCCATCTTCTCCTTATACTGTTTCCTTCTTTATGCAGACAAGATACATCATCGGAAGAAATTTCCTTCGAATGAAAGGTGATCCGTCGATTGTCATATTCTCCATCTTTGGTCAAGGTGTCATGGGTTTAATTCTATCGTCCGTTTTTTACAATTTGCAGCCGACCACCGGCTCCTTCTATTACCGTGGTGCTGCCATGTTTTTTGCTGTGTTGTTCAATGCTTTTGCTTCCTTATTGGAAATCATGTCTTTGTTTGAGGCTCGTCCAATCGTTGAAAAGCATAAAAAGTATGCTTTGTACAGACCTTCTGCTGACGCTCTAGCGTCCATCATCTCGGAACTTCCTGTCAAGCTTTGCATGTCCACGTGTTTCAATTTTTCCTTCTACTTTATGGTTCATTTCAGGCGTGATCCCGGCAGATTCTTTTTCTACTGGCTCTTCTGTGGTCTCTGTACATTGTGTATGTCCCACATGTTTAGGTCCTTGGGAGCTGTGTCTACTTCTCTCGCGGCTGCTATGACTCCTGCTACTTCGGTATTGTTGGCAATGGTTATCTTTACTGGTTTCGTCATTCCCATTCCGTCGATGTTGGGTTGGTGCAGGTGGATTCAATACATCAATCCTGTGTCTTATGTTTTCGAATCATTGATGGTGAACGAGTTCCATGGTCGTAAATTCGAATGTGCTCAATTTGTGCCTTCAGGAGGACCATATGATCAAGTTGCCGCTGTCAACCGTGTGTGCTCAACCGCAGGTGCCAGACCCGGTGAGGATTTTGTTGATGGTACTGCCTATTTGCAGACCTCCTTTGAATATGTGAATGCTCACAAGTGGAGAAATTTGGGTATCGTTGTCGCTTATATCGTCGTCTTTTTGGGCGTCTACATTGCCTTGACCGAATTTAACAAAGGGGCTATGCAAAAGGGTGAGATTGCTTTGTTCTTGAGAGGTTCTTTGAAGAAGGTCAGAAAGCAAAGAGAGCAGAATGAAGCCAAGGTCAATGATGTAGAGAACAACCTTCCAAATGAGAAGATTTCATATTCCGATGCCATGGAGAAGGACTCTGGCGAATCATCTACTTCTGATGACAAACTTCCTAATCAGAGACAGATTTTCCATTGGAAGGATTTGACTTACCAGGTCAAAATCAAGGCAGAAAATCGTGTTATTTTGAATCATGTTGATGGTTGGGTGAAGCCAGGTCAGATCACTGCATTGATGGGTGCTTCTGGTGCTGGTAAGACCACTTTATTGAACTGTTTGTCCGAGCGTCTTACGACTGGTACTGTTACTGACGGTGTAAGAATGGTCAACGGTCACGGTTTGGACTCCTCATTCCAAAGATCGATTGGTTATGTGCAGCAACAAGATATTCATTTGGCAACTTCCACTGTTCGTGAGGCATTGACATTCTCTGCCTACTTAAGACAGCCTTCTCATGTTTCAAAGAAGGAAAAGGACGAATACGTCGACTACGTCATTGATTTGTTGGAGATGGGTGCTTACTCTGATGCTTTGGTTGGTGTTGCTGGTGAGGGTTTAAACGTTGAGCAGCGTAAGAGATTGACAATTGGTGTTGAATTGGTTGCCAAGCCTAAGTTGTTGCTTTTCTTGGATGAGCCTACTTCTGGTTTGGACTCTCAGACTGCTTGGTCTATTTGTAAGTTGATGAGAAAGTTGGCCAACCACGGTCAAGCTATTTTGTGTACCATCCATCAACCTTCAGCTATCTTGTTACAAGAATTCGACCGTTTGTTGTTCTTGCAAAAAGGTGGTAAGACTGTATATTTCGGTGACTTGGGCAAGAACTGTCAAGGTTTAATCGATTACTTTGAGAAGCACGGTGCCCATCCATGTCCTCCAGATGCTAATCCCGCTGAATGGATGTTGGAGGTTGTGGGTGCTGCTCCTGGTTCTAAGGCTGCTCAGGATTACTTCGAAGTTTGGAGAAACTCAGAGGAATACCAGGAAGTTCAGCGTGAATTGGCTTACATGGAAAATGAATTGGGTAAATTGCCTGTGGATGAGGACCCGGAGTCCAGAAAGAAGTACGCCACTTCTCTCATCAAACAGTATTTCATTGTCACTTGGAGAACGTTCCAGCAATATTGGAGAAGCCCTGGTTACATTTATTCGAAGTTTTTCTTAGTTATTACGGCGTCTTTGTTCAATGGTTTTGCCTTCTTCCACAGTGGTACCTCTCAGCAGGGTTTGCAGAATCAAATGTTTTCCATGTTTATGTTTTACATGCCCTTGCAAACATTGATTCAGCAGATGTTGCCATATTACGTTATGCAAAGAGAAATCTATGAAGTGAGAGAAGCACCATCGAGGACATTTAGTTGGTTTGCTTTCATCGCCTCTCAGATTACTACTGAAATTCCTTTCCAGGTTGTTCTTGGTACCGTTGCTTTCTTCTGTTGGTACTACCCTGTTGGACTATACCAAAATGCTACGCCAACTGACACTGTTCATGAGCGTGGTGCTTTAATGTGGTTATTGGTGACTGCGTTTTACGTCTACACAATCTCATTGGGTCAAATGGTTGTTGCTTTCATGGAAATTGCTGACAACGCTGCTAACATGGTCAATTTGATGTTCATCATGTGTTTGAACTTCTGTGGTGTTCTTGCCACTCCGGAGGCATTGCCAGGTTTCTGGATTTTCATGTACAGATGTAACCCATTCACGTACTTGATTCAAGCCATGTTGAGTACTGGTTTGGCCAACACCAAGATCGTGTGCTCTTCTAGAGGAATCTTGCACTTCCAGCCCCCAAGTGGTCAAACTTGTGGCCAGTACATGCAACAATTCATTTCAGCAGCCGGTGGCTACTTGCTTGATGAAAGTGCTACTGATCAGTGTGACTTTTGTGCCATGTCTCAAACAAACACTTTCTTGGATTCCGTCCACGCGGTTTATTCTGAGAGATGGAGAAACTTTGGTATCTTCATCGCTTTCATCGCTATCAACATGATCGGTACCATATTTTTCTACTGGCTTGCAAGAGTGCCAAAATCTTCCAAGTCTAAAAACCACGGCggccgccatcatcaccatcatcatTAA

**Supplementary Table S4.**

**Table S4.** Generation times of strains used in this study.

|  | **Generation time [h] during exponential phase in SD medium pH 6.8** |
| --- | --- |
| Y1857 | 2.10 |
| Y525 | 1.97 |
| Y570 | 2.03 |
| Y2765 | 2.28 |
| Y2766 | 2.29 |
| Y2767 | 1.94 |
| Y2768 | 1.94 |
| Y2769 | 1.96 |

**Supplementary Figure S1.**


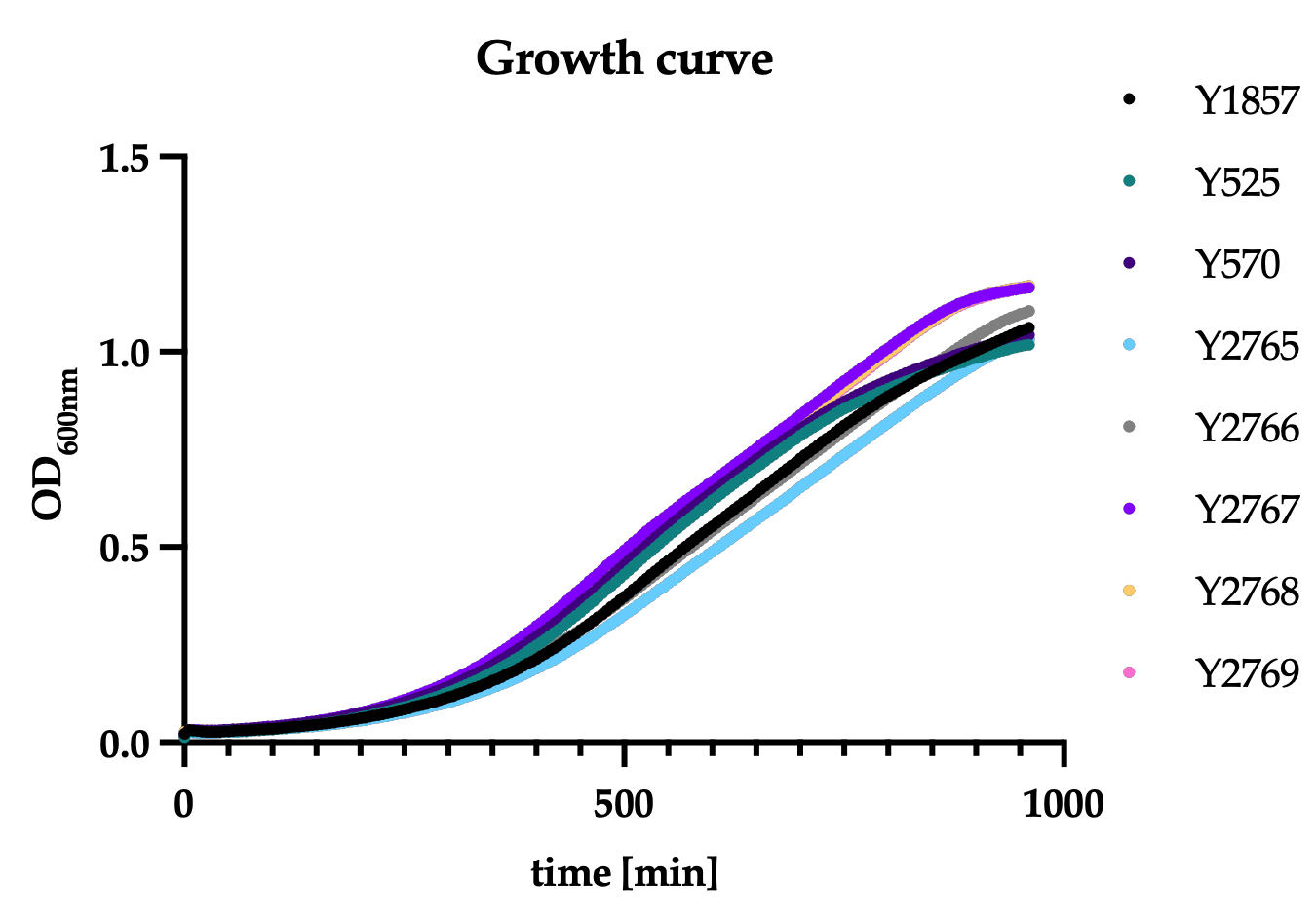


**Figure S1.** Growth curves of strains used in this study. The experiment was caried out in 96-well plates containing buffered SD medium. OD600nm was measured every 5 min during a period of 16 h using a Synergy 2 plate reader at 30°C.

**Supplementary Figure S2.**


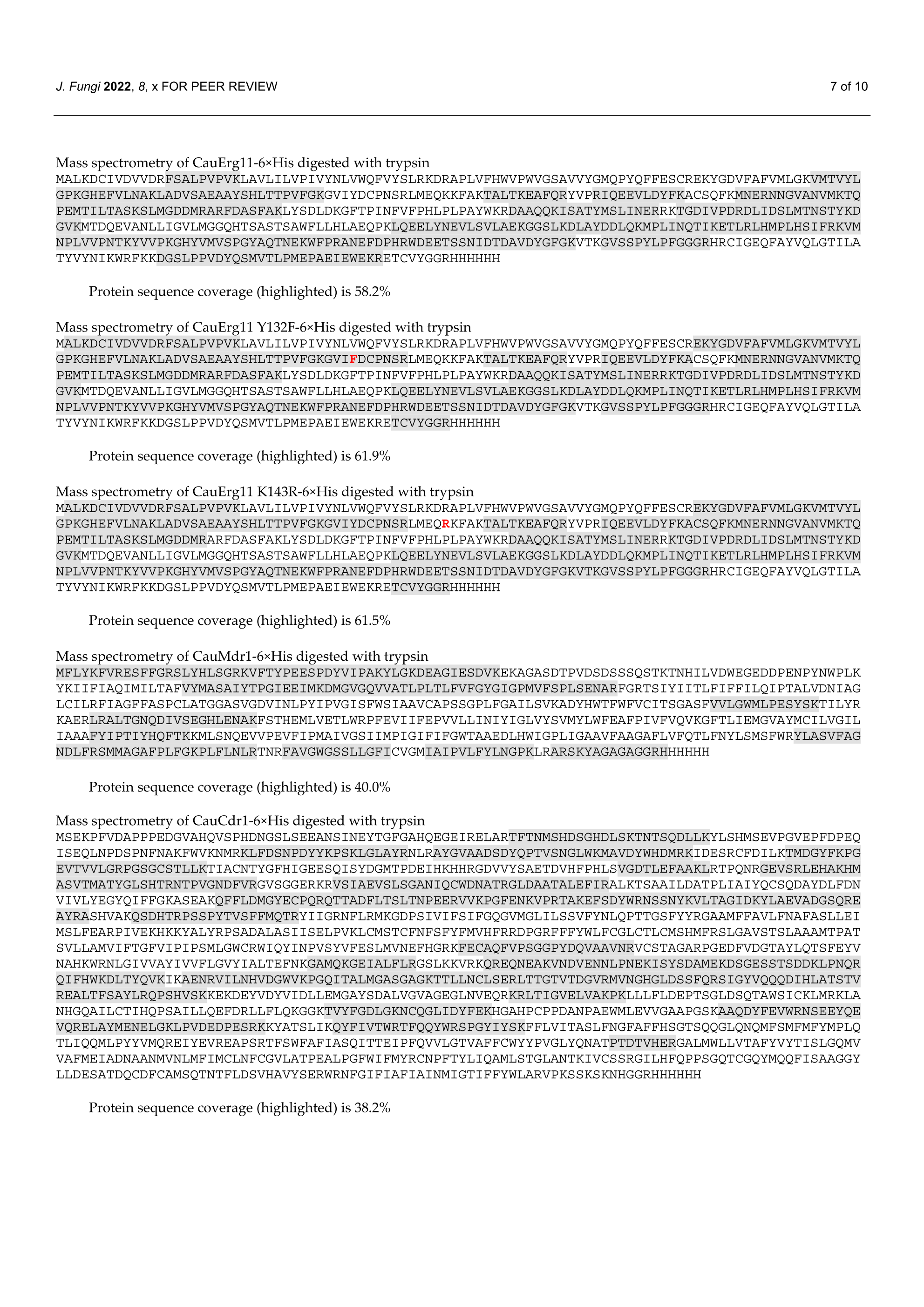


**Figure S2.** Mass spectrometry analysis of tryptic fragments of protein bands.

**Supplementary Figure S3.**


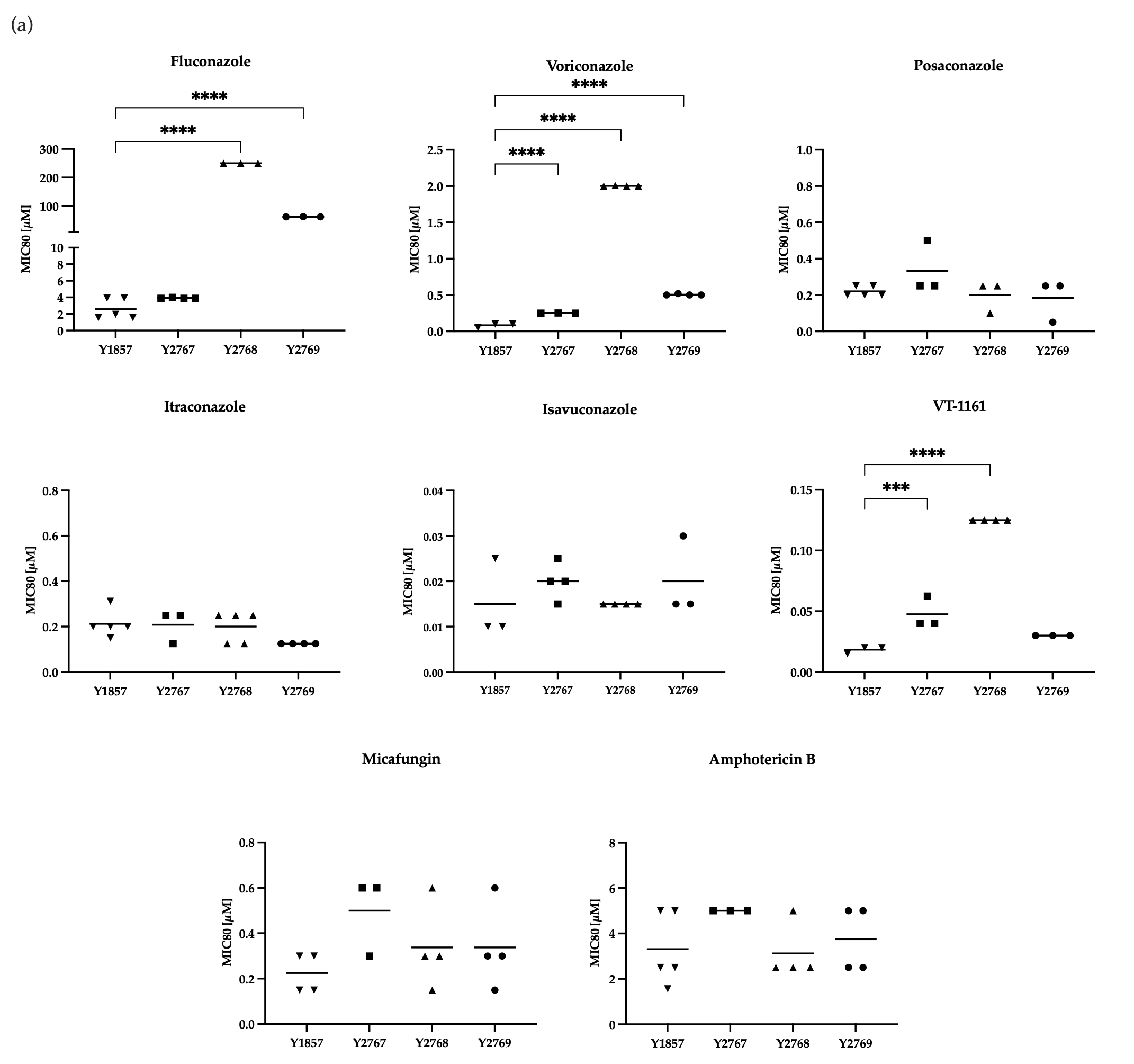


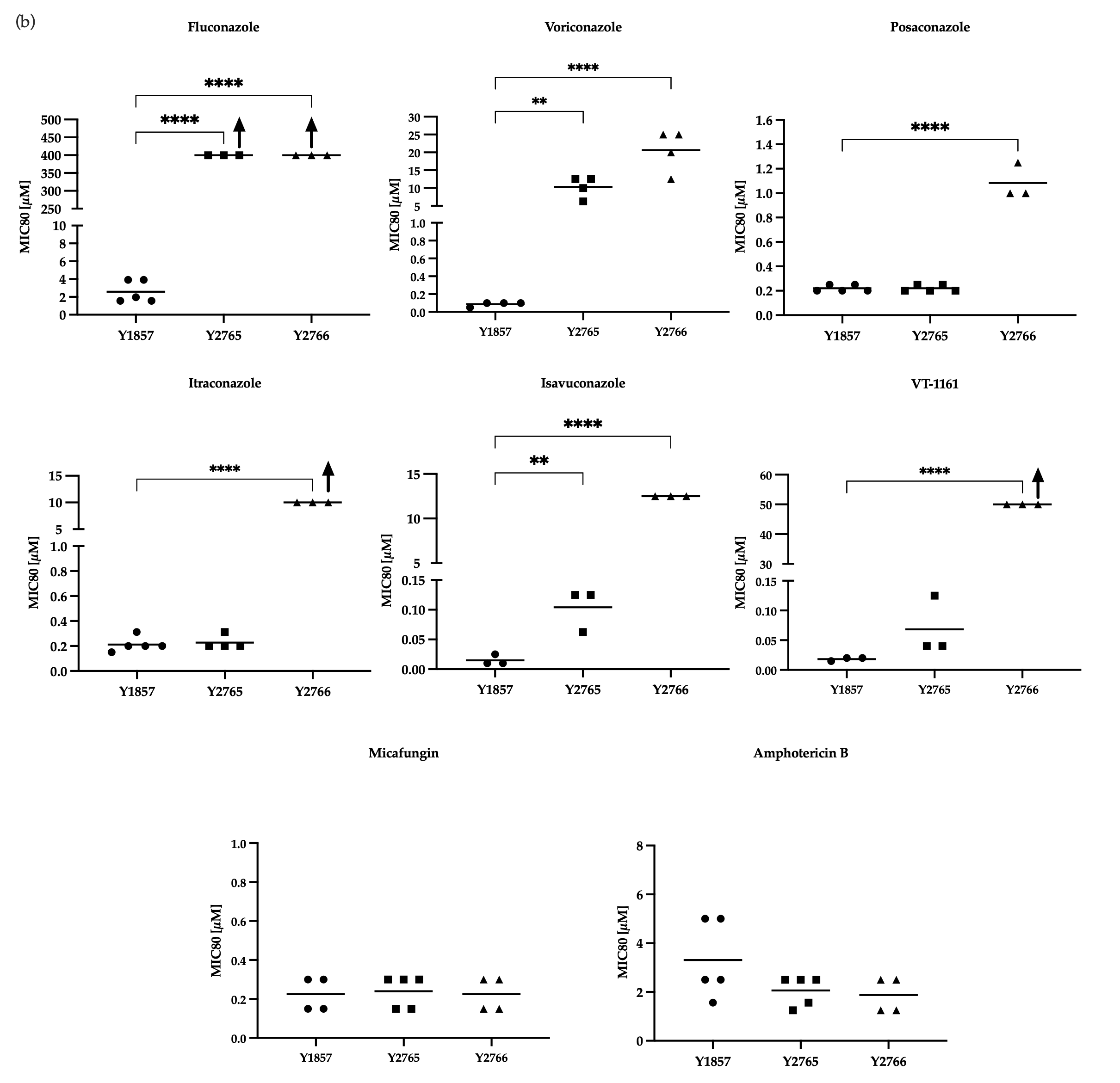


**Figure S3.** Drug susceptibilities of strains used in this study. Strains expressing (**a**) Endogenous ScErg11 (host strain ADΔΔ) or recombinant CauErg11, CauErg1 Y312F or CauErg11 K143R, and (**b**) Endogenous ScErg11 (host strain ADΔΔ), recombinant CauMdr1 or CauCdr1. The strains obtained in this study were tested against the short-tailed azoles FLC and VRC, and the long-tailed azoles POS and ITC, the mid-tailed azole ISA and the tetrazole VT-1161. The polyene AMB and the echinocandin MFG were used as independent controls. MIC_80_ measurements were performed in SD medium buffered at pH 6.8 as described in Materials and methods. The MIC_80_ values were determined after 48 h incubation at 30°C with background subtracted. Statistical analysis was carried out with GraphPad Prism 9 (Graph Pad Software, LLC, CA, USA) using an ordinary one-way analysis of variance (ANOVA) with a post hock Dunnett test. Scatter plot shows results for at least three biological replicates (n=3) with the bars representing the mean value. *p* value 0.0021 (**), 0.0002 (***), < 0.0001 (****). Only data with significant P values are shown in figures. Arrow indicates solubility limit of antifungal reached and an MIC higher than shown.

**Supplementary Figure S4.**


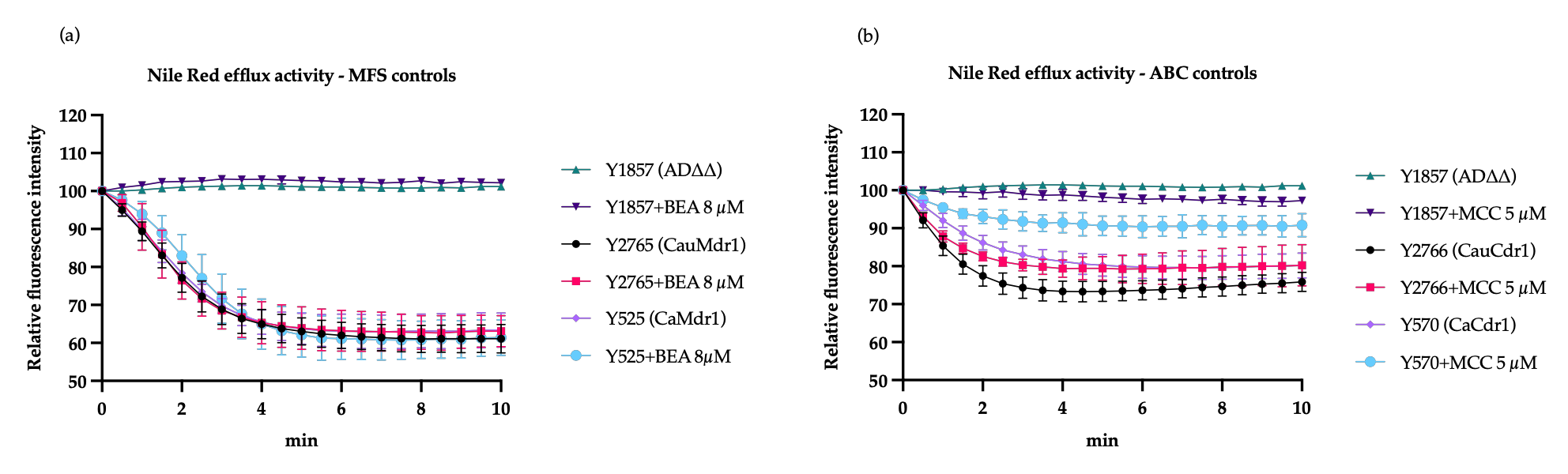


**Figure S4.** Nile Red assays with control compounds. (**a**) MFS pumps activity was not inhibited by 8 µM BEA. (**b**) MCC1189 had no impact on the efflux activity of CauCdr1 (Y2766) but caused a minor inhibition of efflux by CaCdr1B (Y570). (**a**,**b**) The control strain Y1857 had no detectable efflux activity i.e. no reduction in relative fluorescence intensity was observed. The addition of BEA or MCC1189, respectively did not change the fluorescence intensity of cells containing Nile Red. Figures show mean of three biological replicates (n = 3) and bars indicate standard deviation. MCC = MCC1189, BEA = beauvericin.

**Supplementary Figure S5.**


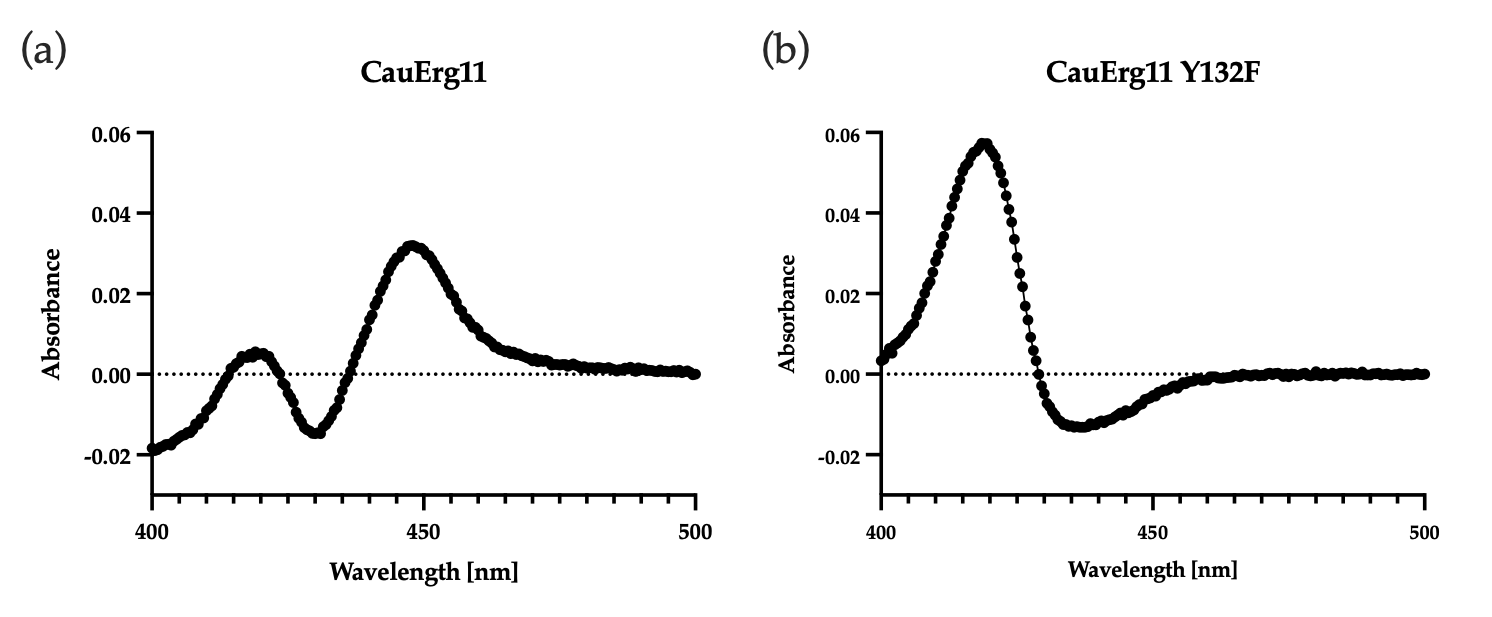


**Figure S5.** Carbon monoxide binding by (**a**) wild-type CauErg11 and (**b**) Y132F mutant. Wild-type CauErg11 gave an expected peak at 450 nm with a smaller shoulder at 418 nm. The Y132F mutant showed no CO binding at 450 nm instead a trough at 435 nm and a peak at 418 nm.
